# Investigation of the Anticancer and Drug Combination Potential of Brominated Coelenteramines toward Breast and Prostate Cancer

**DOI:** 10.3390/ijms232213981

**Published:** 2022-11-12

**Authors:** Carla M. Magalhães, Patricia González-Berdullas, Mariana Pereira, Diana Duarte, Nuno Vale, Joaquim C. G. Esteves da Silva, Luís Pinto da Silva

**Affiliations:** 1 Chemistry Research Unit (CIQUP), Institute of Molecular Sciences (IMS), Department of Geosciences, Environment and Territorial Planning, Faculty of Sciences, University of Porto, Rua do Campo Alegre s/n, 4169-007 Porto, Portugal; 2 OncoPharma Research Group, Center for Health Technology and Services Research (CINTESIS), Rua Doutor Plácido da Costa, 4200-450 Porto, Portugal; 3 CINTESIS@RISE, Faculty of Medicine, University of Porto, Al. Prof. Hernâni Monteiro, 4200-319 Porto, Portugal; 4 Department of Community Medicine, Health Information and Decision (MEDCIDS), Faculty of Medicine, University of Porto, Rua Doutor Plácido da Costa, 4200-450 Porto, Portugal; 5 LACOMEPHI, GreenUPorto, Department of Geosciences, Environment and Territorial Planning, Faculty of Sciences, University of Porto, Rua do Campo Alegre s/n, 4169-007 Porto, Portugal

**Keywords:** coelenteramine, coelenterazine, cancer therapy, drug combination, chemiluminescence, bioluminescence

## Abstract

Cancer is a very challenging disease to treat, both in terms of therapeutic efficiency and harmful side effects, which continues to motivate the pursuit for novel molecules with potential anticancer activity. Herein, we have designed, synthesized, and evaluated the cytotoxicity of different brominated coelenteramines, which are metabolic products and synthesis precursors of the chemi-/bioluminescent system of marine coelenterazine. The evaluation of the anticancer potential of these molecules was carried out for both prostate and breast cancer, while also exploring their potential for use in combination therapy. Our results provided further insight into the structure–activity relationship of this type of molecule, such as their high structural specificity, as well highlighting the 4-bromophenyl moiety as essential for the anticancer activity. The obtained data also indicated that, despite their similarity, the anticancer activity displayed by both brominated coelenteramines and coelenterazines should arise from independent mechanisms of action. Finally, one of the studied coelenteramines was able to improve the profile of a known chemotherapeutic agent, even at concentrations in which its anticancer activity was not relevant. Thus, our work showed the potential of different components of marine chemi-/bioluminescent systems as novel anticancer molecules, while providing useful information for future optimizations.

## 1. Introduction

Cancer is one of the most challenging diseases currently faced by humanity; in 2018 alone, 17 million cases were reported worldwide, leading to 9.6 million deaths [1]. Despite the development of targeted therapies and more effective chemotherapy agents, many patients still lack effective cancer treatment [2]. Moreover, common approaches (such as chemotherapy) can present insufficient selectivity and cause severe side effects [3,4]. Even more selective approaches, such as targeted therapy and immunotherapy, can also induce some side effects (e.g., skin problems or autoimmune reactions), while not being useful for all patients (who lack the target for the therapy) [5]. Given this, it is essential that new therapeutic agents are developed to be more effective and safer for patients.

**Scheme 1:**
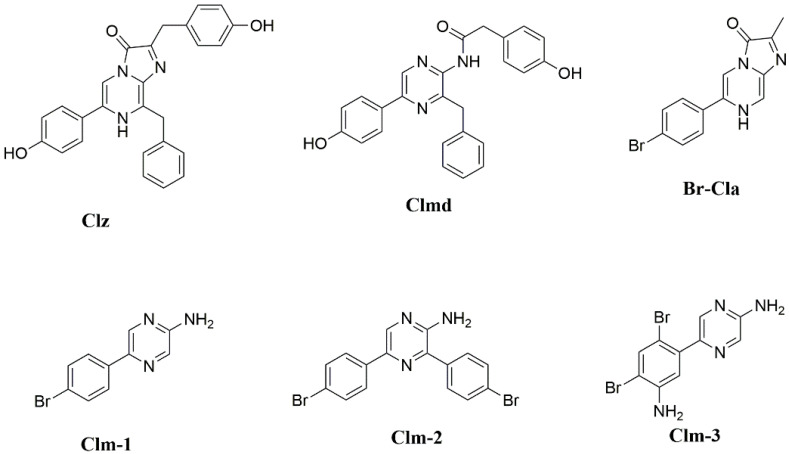
Structures of naturally occurring coelenterazine (**Clz**) and coelenteramine (**Clm**), and their synthetic derivatives **Br-Cla** and **Clm-1-3**.

Some members of this team have been active in recent years in the development of novel strategies for cancer therapy based on the chemiluminescent system of marine coelenterazine (**Clz**) (Scheme 1) [6,7,8,9]. Chemiluminescence consists of the conversion of thermal energy into excitation energy, due to a chemical reaction, leading to light emission [10,11]. For **Clz**, its chemiluminescent reaction can be triggered solely by superoxide anions, a reactive oxygen species (ROS) that is usually overexpressed in tumor cells [12,13]. More specifically, the reaction with superoxide anions allows for the oxygenation of the imidazopyrazinone core of **Clz**, with the formation of a high-energy cyclic peroxide intermediate (dioxetanone). This latter compound decomposes readily into the chemiexcited chemiluminophore, coelenteramide [10,11]. Besides chemiluminescence, **Clz** is also capable of bioluminescence in many marine organisms, in combination with either luciferase enzymes or photoproteins [10,11,14].

Specifically, our objective is to use the **Clz** system to develop single-molecule and self-activating photosensitizers to overcome the current limitations of photodynamic therapy (PDT) [6,7,8,9]. PDT is a clinically approved cancer therapy with a minimally invasive nature, few side effects, and a fast-healing rate for healthy tissues [15,16]. In PDT, a photosensitizer is administered to the patient and accumulates in tumor tissue; then, it is activated by light irradiation to sensitize the cytotoxic singlet oxygen [15,16]. While PDT is selective and has relatively broad-spectrum applications in terms of tumor types, the low penetration of light into the tissue limits its use in the treatment of more superficial tumors [17,18]. So, removing this limitation of PDT would allow more widespread use of this cancer therapy, while maintaining its relevant advantages.

The chemiluminescent system of marine **Clz** has potential to address the restrictions of PDT as the decomposition of the high-energy dioxetanone intermediate allows for the direct chemiexcitation from the singlet ground state to excited states [6,7,8,9], without light irradiation. More specifically, the direct chemiexcitation to triplet states should allow the intracellular generation of the cytotoxic singlet oxygen through interaction with molecular oxygen. Furthermore, this can be achieved with tumor selectivity, as this process is triggered by a general cancer marker (overexpressed superoxide anion) [6,7,8,9,10,11].

In this context, members of this team have developed different single-brominated **Clz** analogs (**Cla** compounds) [6,7,8,9], with addition of bromine heteroatoms being made for enhancing triplet-state chemiexcitation via the heavy-atom effect [6,7,8,9]. These compounds showed anticancer potential toward different cancer cell lines (breast, prostate, lung, and gastric cancer) [6,7,8,9]. Moreover, while the addition of bromine was found to be essential to their anticancer activity, the specific position of his halogen was not found to be relevant [6,7,8,9,10,11]. Among different **Cla** compounds, **Br-Cla** (Scheme 1) was initially developed as a proof of concept, as it showed singlet oxygen sensitization in solution due to a superoxide-anion-induced chemiluminescent reaction [6]. Moreover, it showed anticancer activity toward both prostate and breast cancer (IC_50_ values of 24.3 and 21.6 µM, respectively) [6,8], while having no effect on the cellular viability of noncancer cells at the same concentration range [6]. Thus, this type of compound showed an interesting anticancer activity and a relevant profile of safety, thereby showing potential as a starting point for future optimization in light-free PDT.

Following this, we have recently expanded our research of brominated analogs of molecular components of the **Clz** chemi-/bioluminescent system (**Br-Cla** included), with focus on their activity towards both gastric and lung cancer in vitro [9]. Among the studied compounds, we also included a brominated analog of marine coelenteramine (**Clm-1**, Scheme 1) [9]. Coelenteramines are both metabolic products of bioluminescent systems involving **Clz** [19] and intermediates in the chemical synthesis of **Clz** and analogs [6,7,8,9,11]. Relevantly, they comprise an aminopyrazine core, instead of an imidazopyrazinone one, meaning that they are incapable of undergoing a chemiluminescent reaction. Despite this, we have unexpectedly found that **Clm-1** showed higher anticancer activity than **Br-Cla** and other brominated **Cla** compounds, reaching IC_50_ values of 15.2 and 32.6 µM for gastric and lung cancer cell lines, respectively [9]. Given the structural similarities between **Br-Cla** and **Clm-1** (Scheme 1), the higher potency of the latter toward lung and gastric cancer cell lines, and the fact that it cannot undergo a chemiluminescent reaction, some doubts arose regarding the mechanism of action of both of the compounds. That is, if **Br-Cla** and **Clm-1** share the same mechanism of action, and the former is just a less potent analog of the latter, then the anticancer activity of **Br-Cla**-like compounds cannot be due to a self-activating photodynamic effect. That is, this family of compounds could be instead a new type of chemotherapeutic agent. If **Br-Cla** and **Clm-1** do not present the same mechanism of action, this would lead to the exciting conclusion that bromination of different components of the **Clz** chemi-/bioluminescent systems produces different types of molecules with therapeutic potential. The elucidation of this matter is essential for the full comprehension of the potential of the **Clz**-based system in cancer therapy and how to optimize its anticancer activity.

Herein, to help to clarify this topic, for the first time, we investigated the in vitro cytotoxicity of **Clm-1** toward both prostate (PC-3) and breast (MCF-7) cancer cell lines. These were cell lines used previously in our initial evaluation of the anticancer activity of **Br-Cla** [6]; so, their use here will allow us to further assess the relative potency of **Br-Cla** and **Clm-1** in cell lines other than those of lung and gastric cancer [9]. Additionally, we have also synthesized and investigated two new **Clm-1** analogs: **Clm-2** and **Clm-3** (Scheme 1). In the study where we discovered the anticancer activity of **Clm-1** [9], we also found that it is linked to the presence of the 4-bromophenyl moiety at the *para*-position of the aminopyrazine core. Thus, the two new analogs were designed to obtain further insight into structure–activity relationship of these compounds. More specifically, **Clm-2** was obtained to assess whether the inclusion of an additional 4-bromophenyl moiety (Scheme 1), this time at the *ortho*-position of the aminopyrazine core, helps to increase the anticancer activity. In turn, **Clm-3** was designed to verify how sensitive **Clm-1** is to the introduction of different functional groups to the 4-bromophenyl moiety at the *para*-position.

Finally, we have also evaluated the potential for this type of compound to be used in combination therapy, in which more than one molecule or modality is used to treat a single disease [20,21]. The combination of molecules/modalities can present relevant advantages, such as requiring lower dosages for achieving an equal or even higher level of efficiency, which can be important in reducing the side effects of therapies [22]. Thus, we also investigated the cytotoxicity of the best-performing **Clm** compound in combination with a known chemotherapeutic agent. 

## 2. Results and Discussion

### 2.1. Synthesis and Structural Characterization of ***Clm-1**–**Clm-3***

**Clm-1**–**Clm-3** (Scheme 1) were synthesized with methodologies already validated and optimized by our team [6,7,8,9,11], which are further detailed in the Supporting Information. In short, they consist of Suzuki–Miyaura cross-coupling between commercial 5-bromopyrazin-2-amine (**Clm-1** and **Clm-3**) or 3,5-dibromopyrazin-2-amine (**Clm-2**) and an appropriated phenylboronic acids (either 4-bromophenylboronic acid or 3-aminophenylboronic acid). This type of reaction already yields both **Clm-1** and **Clm-3**. For obtaining **Clm-2**, an additional reaction between *N*-bromosuccinimide and 5-(3-aminophenyl)pyrazin-2-amine (the product of the previous step) in ethanol is needed. The structures of compounds and synthesis intermediates were confirmed with both ^1^H/^13^C NMR and FT-MS spectroscopy (Figures S4–S18).

### 2.2. Photophysical Characterization of ***Clm-1**–**Clm-3***

The next step of this study was to perform the photophysical characterization of **Clm-1** and **Clm-3**, by both UV-Vis and fluorescence spectroscopy. While not being expected to be directly related with the anticancer activity of this type of compounds, their photophysical characterization should be of interest nonetheless. Firstly, it allows us to determine whether their structural differences lead to significant changes to their intrinsic (photophysical) properties. Additionally, by analyzing their absorption/fluorescence in different media, we can observe how sensitive these compounds are to changes in the local microenvironment, and how differently they behave in relation to each other. This type of information could provide some potentially relevant insight, especially if we consider their mechanism of action as binding to an active/allosteric site of a yet-to-be-identified target.

**Figure 1:**
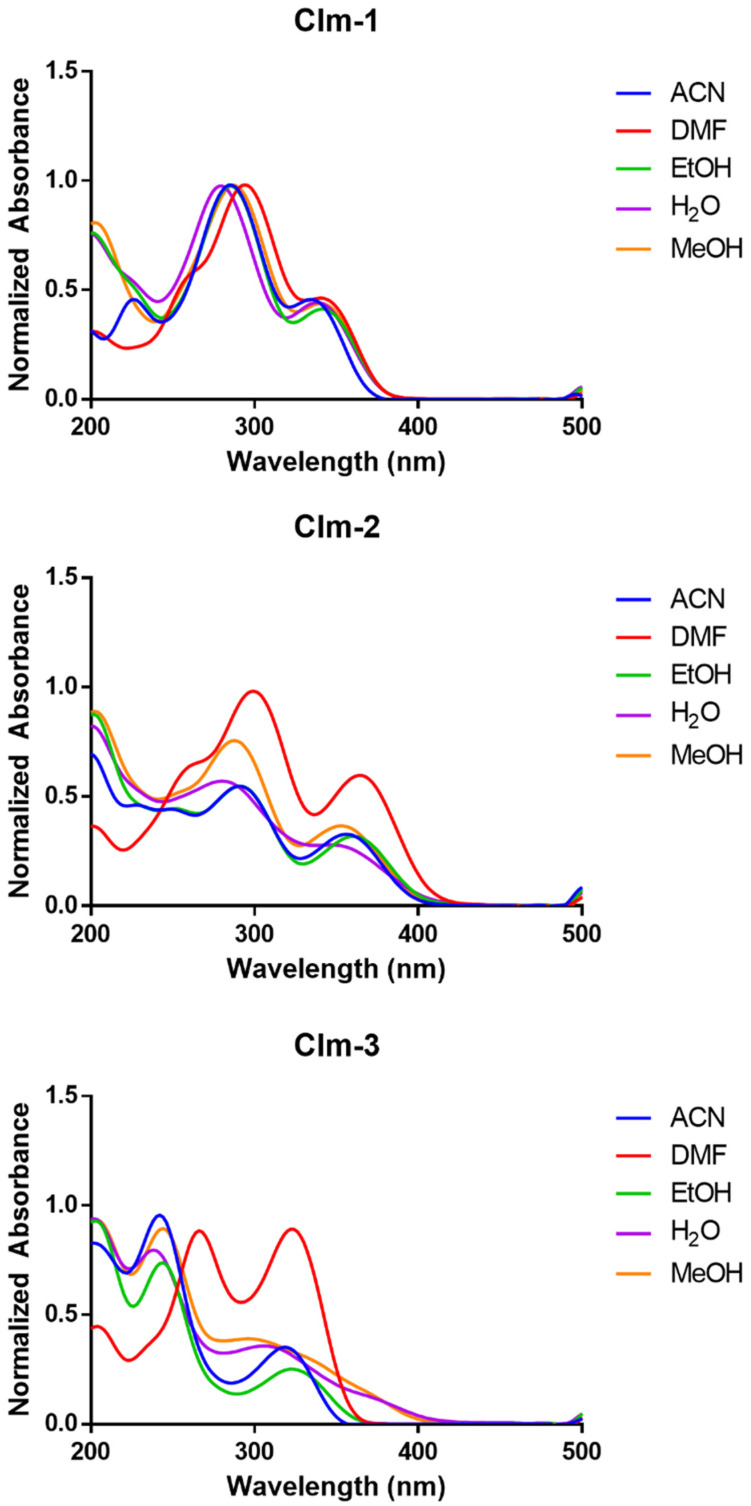
Normalized absorption spectra of **Clm-1-3** in protic (H_2_O, EtOH, and MeOH) and aprotic (ACN and DMF) solvents, with final concentration of 30 µM for all compounds.

So, we started by measuring the UV-Vis absorption spectra of these compounds in protic (H_2_O, MeOH, EtOH) and aprotic (ACN and DMF) media (Figure 1). All compounds present spectra with similar shape, with a more intense and blue-shifted band, and with a less intense and red-shifted peak. More specifically, **Clm-1** presents a more intense band at ~280–290 nm, and a less intense band at ~340 nm. No particularly relevant difference between solvents was found. In the case of **Clm-2**, there is an average ~10 nm red-shift for the first band (~290–300), and a ~20 nm red-shift to ~360 nm for the second. Once again, no relevant effect exerted by the solvent was found. Finally, **Clm-3** does present more blue-shifted absorption, at both ~240–260 nm and ~310–320 nm, without any relevant effect from the solvent. Thus, these results indicate that only the structural modifications exert some effect on the UV-Vis absorption of these compounds. However, there is limited impact on the absorption wavelength maxima. The exception is just in DMF for **Clm-2** and **Clm-3**.

**Figure 2:**
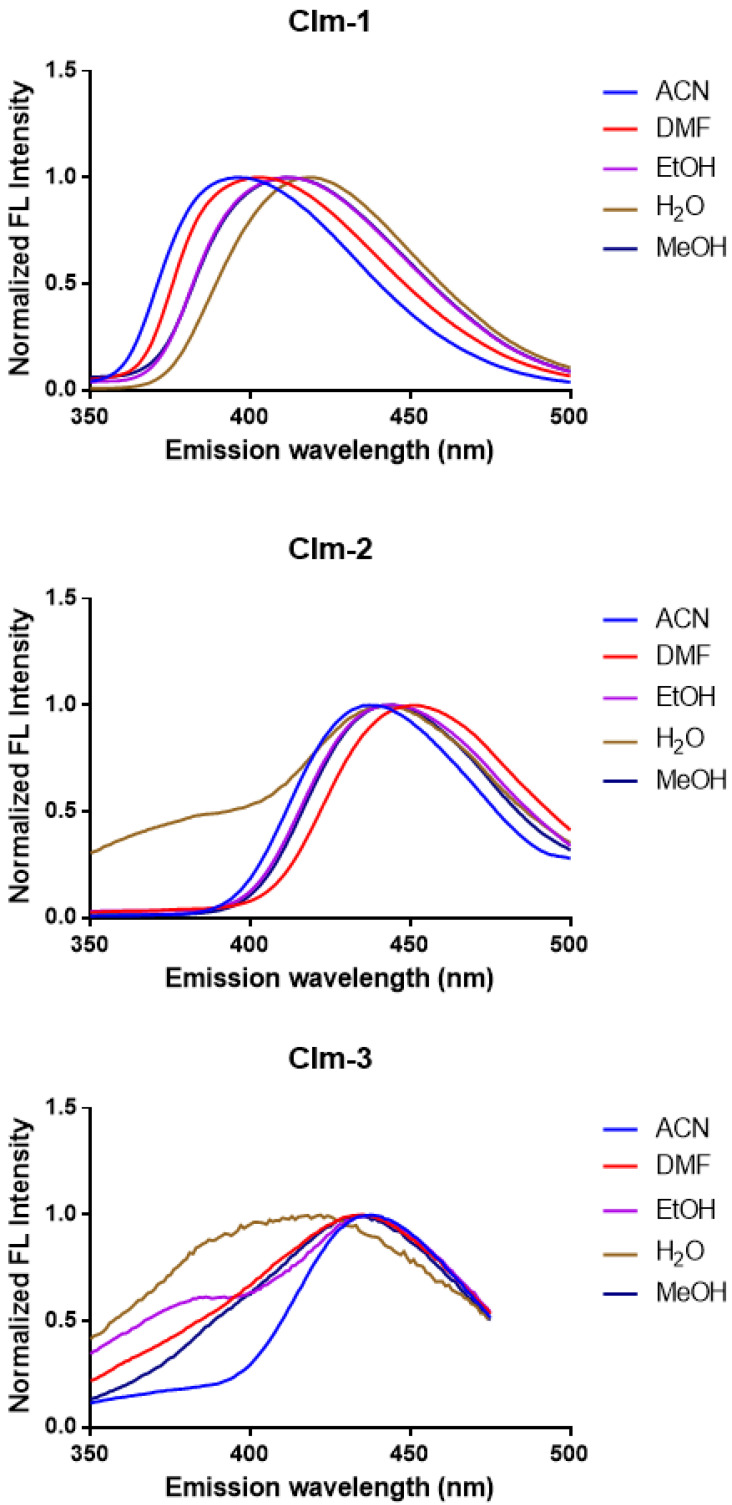
Normalized fluorescence spectra of **Clm-1-3** in protic (H_2_O, EtOH, and MeOH) and aprotic (ACN and DMF) solvents, with final concentration of 30 µM for all compounds.

To gain further insight in the properties of these **Clm** molecules, the fluorescence emission spectra (Figure 2) and the 2D excitation–emission matrix (EEM) contour plot (Figures S1–S3) were obtained. The EEM plots showed that all compounds emit light with maxima in the ~400–450 nm, with large excitation wavelength ranges (which are associated with the two absorption bands in each spectrum, as seen in Figure 1). Interestingly, while **Clm-1** and **Clm-2** showed only one well-defined emission spectrum, with a broad excitation, **Clm-3** shows instead two well-defined emission centers with the same emission wavelength maxima. Thus, the introduction of additional bromine and amine groups to the 4-bromophenyl moiety at the *para*-position has more impact on their fluorescence than the addition of a second 4-bromophenyl moiety to the *ortho*-position.

As for individual fluorescence spectra (Figure 2), **Clm-1** presents an emission maximum at ~400 nm for ACN and DMF, which becomes increasingly red-shifted in EtOH/MeOH (by ~10 nm) and in H_2_O (by ~20 nm). In turn, **Clm-2** is less sensitive to the solvent, and it presents an emission wavelength maximum of ~440–450 nm, irrespective of the solvent. A similar case is found for **Clm-3**, with emission maxima at ~440–450 nm. Interestingly, both **Clm-2** and **Clm-3** present a shoulder in emission spectra (Figure 1) in some of the studied solvents. **Clm-2** presents a shoulder before the main band emission in H_2_O; meanwhile, for **Clm-3**, the same behavior is observed in CAN and EtOH. Thus, these compounds do appear to be sensitive to changes to the local microenvironment. Moreover, in terms of fluorescence, the chosen modifications to the **Clm-1** structure did result in some relevant differences, as both **Clm-2** and **Clm-3** present with red-shifted emission (by ~30–50 nm). Thus, these modifications do somewhat affect the properties of these compounds. It should be noted that, for **Clm-1**, there are some minor shifts in wavelength maxima from aprotic to protic solvents, and there are further shifts from MeOH/EtOH to H_2_O. This indicates some effect of hydrogen bonding on the fluorescence of **Clm-1**, which is not detected for the other compounds. Thus, **Clm-1** might be able to participate in a different network of hydrogen bonding than the other compounds, which might be relevant in terms of binding to possible targets.

### 2.3. Evaluation of the Cytotoxicity of ***Clm***-Based Compounds towards MCF-7 and PC-3 Cancer Cells

To understand the anticancer activity of **Clm** compounds, and how they actually differ from **Br-Cla**, we evaluated their in vitro cytotoxicity toward both breast MCF-7 and prostate PC-3 cancer cell lines. Namely, we evaluated their toxicity with increasing concentrations, while changes in cell morphology were observed by microscopy.

**Figure 3:**
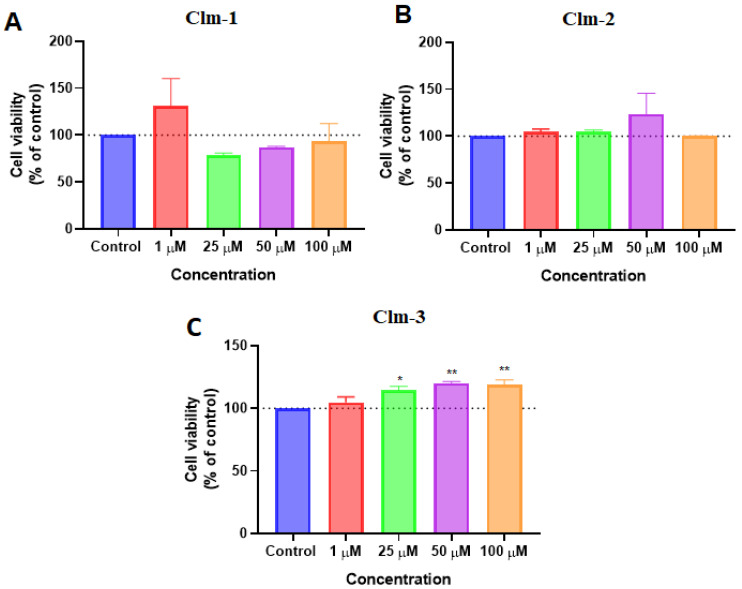
Cytotoxicity evaluation of compounds (**A**) **Clm-1**, (**B**) **Clm-2**, and (**C**) **Clm-3** in MCF-7 cells. MCF-7 were treated with vehicle (methanol) and increasing concentrations (0–100 µM) of each compound for 48 h and cell viability was assessed by MTT assay. Data are given as the mean ± SEM (*n* = 3), * statistically significant vs. control (vehicle) at *p* < 0.05; ** statistically significant vs. control (vehicle) at *p* < 0.01.

First, we analyzed their effect toward MCF-7 breast cancer cells in terms of cytotoxicity (Figure 3) and effect on cell morphology (Figure S11). It should be remembered that all previous **Cla** compounds developed by us were active toward this cell line [6,7,8,9], with **Br-Cla** presenting an IC_50_ of 21.6 µM [8]. Interestingly, none of the compounds showed activity toward this cancer cell line, which was an opposing result to the findings for **Br-Cla** and other **Cla** compounds [6,7,8]. Furthermore, morphological analysis did not show modifications in the morphological structure of the MCF-7 cells.

**Figure 4:**
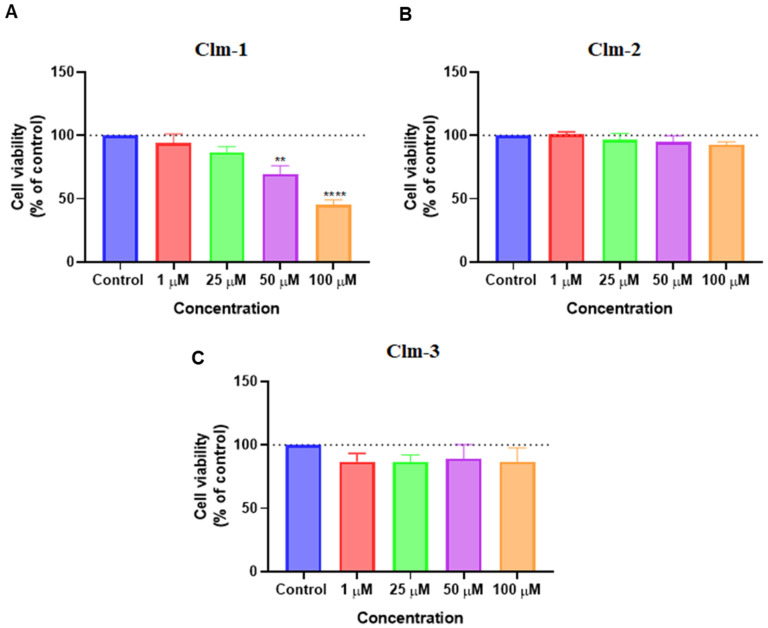
Cytotoxicity evaluation of compounds (**A**) **Clm-1**, (**B**) **Clm-2**, and (**C**) **Clm-3** in PC-3 cells. PC-3 were treated with vehicle (methanol) and increasing concentrations (0–100 µM) of each compound for 48 h and cell viability was assessed by MTT assay. Data are given as the mean ± SEM (*n* = 3), ** statistically significant vs. control (vehicle) at *p* < 0.01; **** statistically significant vs. control (vehicle) at *p* < 0.0001.

The next step was to evaluate the cytotoxicity (Figure 4) and changes in cellular morphology (Figure S12) toward the PC-3 prostate cancer cell line. It should be remembered (once again) that all previously studied **Cla** compounds showed activity toward this cell line [6,7], with **Br-Cla** presenting an IC_50_ value of 24.3 µM [6,8]. Interestingly, **Clm-1** did show some activity by reducing the cellular viability by ~30 and ~50% at higher concentrations of 50 and 100 µM, respectively. By their turn, both **Clm-2** and **Clm-3** showed no anticancer activity. This is supported by microscopic analysis (Figure S12), which revealed that **Clm-2/3** had no effect on the morphological structure of these cells. On the contrary, **Clm-1** induced the formation of aggregates at a concentration of 25 µM and at higher concentrations (50 and 100 µM), in which was visible in the change in the size and shape of the cells, which were larger and longer. Compared with conventional chemotherapeutic drugs, such as doxorubicin or paclitaxel, we have previously demonstrated that these drugs have IC_50_ values of 0.17 µM [23] and 0.1 µM [24] for breast and prostate cancer cells, respectively. Therefore, it can be concluded that these newly synthesized compounds have a much lower anticancer potential than the antineoplastic agents commonly used for these types of cancer.

**Figure 5:**
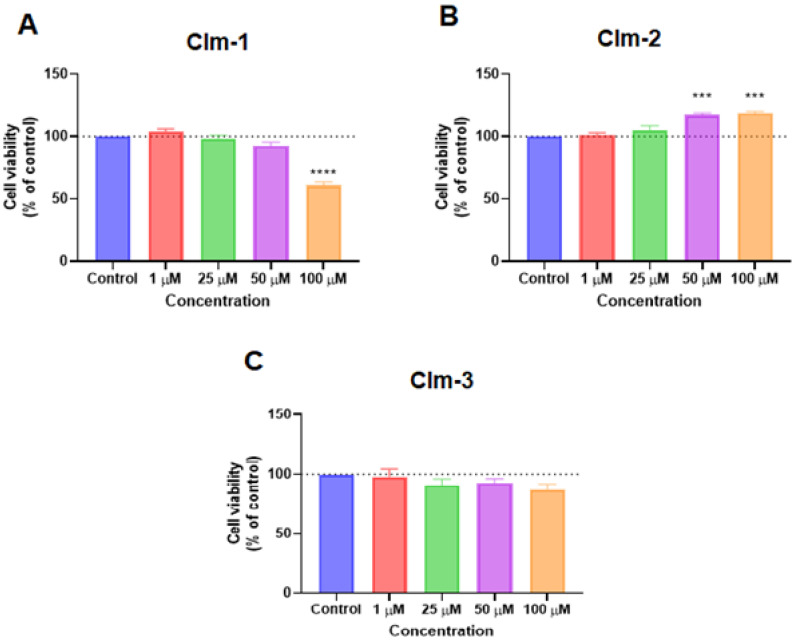
Biosafety evaluation of compounds (**A**) **Clm-1**, (**B**) **Clm-2,** and (**C**) **Clm-3** in MRC-5 cells. MRC-5 were treated with vehicle (methanol) and increasing concentrations (0–100 µM) of each compound for 48 h and cell viability was assessed by MTT assay. Data are given as the mean ± SEM (*n* = 3), *** statistically significant vs. control (vehicle) at *p* < 0.001; **** statistically significant vs. control (vehicle) at *p* < 0.0001.

Finally, we also assessed the safety of this type of compounds, by evaluating their cytotoxicity toward noncancer MRC-5 cells. As presented in Figure 5 and Figure S13, **Clm-2/3** are clearly molecules without cytotoxic potential for normal cells, as they have no effect on the cellular viability (even at high 100 µM concentrations). However, the same cannot be said for **Clm-1**, in which a concentration of 100 µM induces toxicity of ~40% in MRC-5 cells. It should be noted that **Br-Cla** did not show any effect on the cellular viability of noncancer cells [6]. For one, this result indicates that **Clm-1** has higher activity toward a noncancer cell line than for a cancer one (breast MCF-7), while having comparable activity toward prostate PC-3 cancer cell line. However, it should also be noted that the toxicity toward the noncancer cell line was only observed at a concentration (100 µM) relevantly higher than its IC_50_ toward both gastric and lung cancers (15.2 and 32.6 µM) [9]. Thus, **Clm-1** do show some potential as a compound worthy of optimization for future use in treatment of gastric and/or lung cancer.

Despite the lack of relevant anticancer activity toward breast/prostate cancer cell lines, the results obtained for **Clm-1-3** provide useful information for understanding the anticancer potential of the brominated **Clm** and **Cla** compounds. One of the conclusions is that despite their structural similarities, **Clm-1** and **Br-Cla** present quite different behavior. That is, while **Clm-1** is more potent toward lung and gastric cancer than **Br-Cla** [9], their relative activity is clearly reversed in breast and prostate cancer. Moreover, while Br-**Cla** is active (with variable efficiency) toward these four cancer types, **Clm-1** is inactive toward breast cancer. Thus, these inconsistencies between the anticancer activity of these two compounds toward different cancer cell lines indicate that **Clm-1** and **Br-Cla** should be considered as independent anticancer molecules with different mechanisms of action. This should mean that (i) **Br-Cla** can still be considered as a prototypical system for light-free and self-activating photosensitizers [6,7,8], and (ii) the chemi-/bioluminescent systems of **Clz** appear to be an interesting and unexpected source of different compounds with anticancer potential (when subjected to bromination).

The results toward both PC-3 and MCR-5 cell lines also allow us to conclude that the cytotoxic potential of **Clm-1** is particularly inflexible toward structural modifications. While our previous study identified the 4-bromophenyl moiety as essential to its activity [9], the present results showed that the additional modifications to the aminopyrazine core that still maintain this moiety (**Clm-2**) are enough to impair its anticancer activity. The same can be said by further substitution of the 4-bromophenyl moiety, by inclusion of additional bromine and amino groups, even while maintaining a bromine heteroatom at C_4_ (**Clm-3**). Thus, the mechanism of action of **Clm-1** is clearly specific and sensitive to this 3-(4-bromophenyl)pyrazin-2-amine structure.

### 2.4. Evaluation of the Combination Potential of ***Clm-1*** toward MCF-7 Breast and PC-3 Prostate Cancer Cells

**Figure 6:**
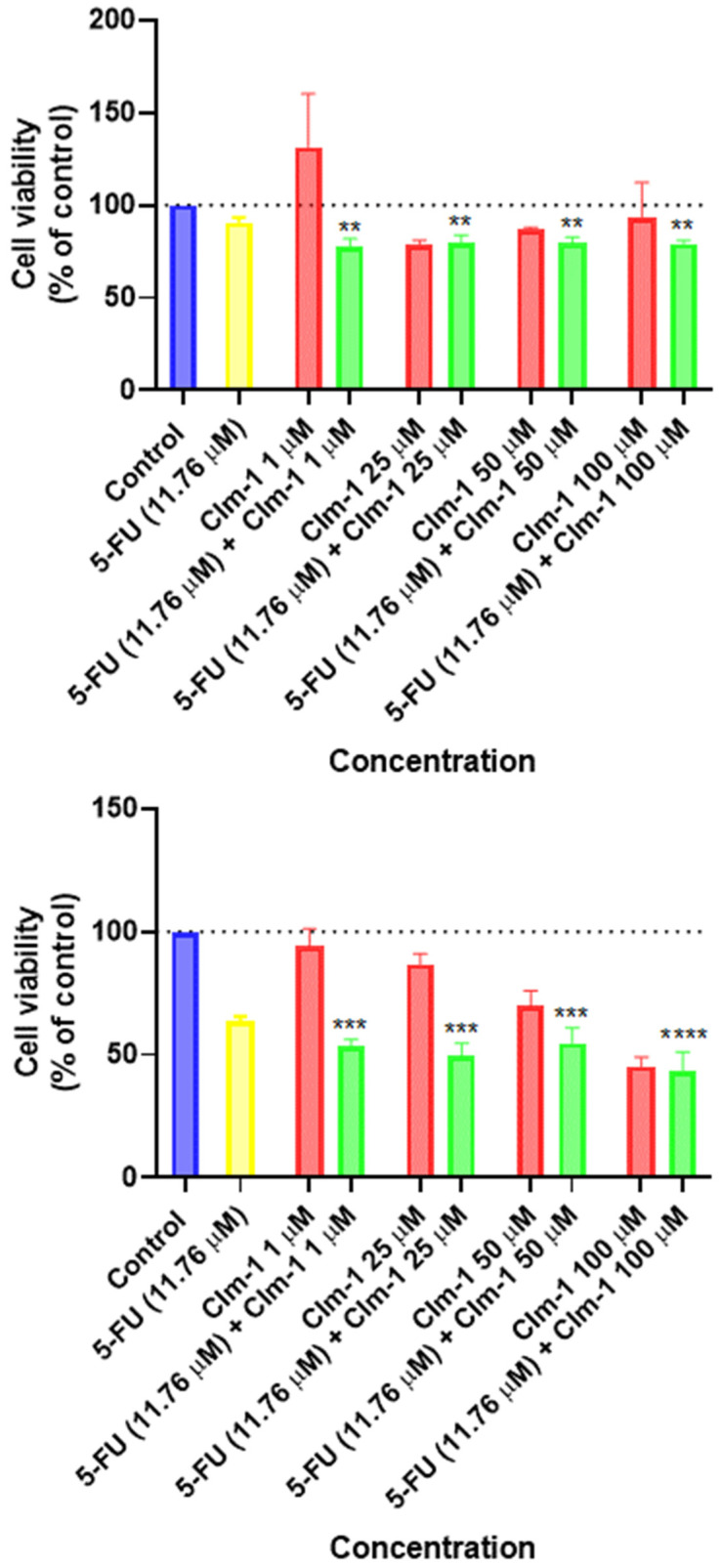
Cytotoxicity evaluation of the combination of 5-FU and different concentrations of **Clm-1** in MCF-7 (top) and PC-3 (bottom) cancer cells. These were treated with vehicle (methanol and DMSO) and with a combination of 5-FU (11.79 µM) and increasing concentrations (0–100 µM) of **Clm-1** for 48 h; cell viability was assessed by MTT assay. Data are given as the mean ± SEM (*n* = 3), ** statistically significant vs. control (vehicle) at *p* < 0.01; *** statistically significant vs. control (vehicle) at *p* < 0.001. **** statistically significant vs. control (vehicle) at *p* < 0.0001.

Finally, we proceeded to test the potential of **Clm-1** (as the best-performing compound) in combination therapy [20,21,22]. More specifically, we evaluated how increasing concentrations of **Clm-1** could affect the anticancer activity of the known chemotherapeutic agent 5-fluorouracil (5-FU) toward both MCF-7 breast and PC-3 prostate cancer cell lines, at the IC_50_ concentration of 5-FU (11.76 µM). Cellular viability was assessed with the standard MTT assay for an exposure period of 48 h, and the results are presented in Figure 6. The analysis of changes in cell morphology can be found in Figures S14 and S15.

Starting with MCF-7 breast cancer cell lines (Figure 6), increasing the concentration of **Clm-1** does not lead to relevant variations in cellular viability. This is expected, as this compound did not show anticancer activity toward this cancer type. However, the combination of this molecule with 5-FU does lead to some improvement in the profile of this chemotherapeutic agent. This is particularly interesting due to the lack of activity of **Clm-1** and due to this improvement being seen already at concentrations as low as 1 µM (significantly lower than the concentration at which this compound is active toward noncancer cells).

Similar results were also observed for PC-3 prostate cancer cell lines (Figure 6 and Figure S15), in which increasing the amount of **Clm-1** does little for decreasing the cellular viability of the PC-3 cell line. However, there is now a somewhat relevant decrease at the higher concentration of **Clm-1**, which can be ascribed to its activity toward PC-3 cancer cells (Figure 4). More importantly, once again, the addition of **Clm-1** improves the profile of 5-FU, even at lower concentration of 1 µM.

These are interesting results, given that **Clm-1** can improve the profile of a known chemotherapeutic agent, even at concentrations/cell lines in which it is not active by itself. Moreover, this improvement is seen at concentrations significantly lower than the ones in which this compound is not safe toward noncancer cells. Thus, this compound shows potential for improving the profile of 5-FU. More specifically, it shows potential to decrease the concentration of 5-FU, which is required to reach its IC_50_. This is of relevance due to the common side effects of chemotherapy, which are potentiated by higher concentrations of the used drug. Therefore, the ability to reduce doses while maintaining activity is a good strategy for reducing side effects without affecting the efficiency of a given treatment. The potential of combining these compounds should be further explored in the future and is a relevant feature of **Clm-1**.

## 3. Materials and Methods

### 3.1. Synthesis of ***Clm*** Compounds

The synthesis of **Clm-1** was performed by employing synthetic routes which have already validated by our team [6,7,8,9], while **Clm-2** and **Clm-3** were synthesized through the same synthetic pathway with some modifications. The detailed synthetic procedure is available in the Supplementary Information. Briefly, these compounds were obtained through a Suzuki–Miyaura crossed-coupling between 5-bromopyrazin-2-amine (**Clm-1** and **Clm-3**) or 3,5-dibromopyrazin-2-amine (**Clm-2**) and the corresponding arylboronic acid derivative using bis(triphenylphosphine)palladium(II) dichloride as the palladium source and potassium carbonate as the base. This reaction yields both **Clm-1** and **Clm-2**. To obtain **Clm-2**, an additional halogenation reaction is required. Namely, the reaction between *N*-bromosuccinimide and 5-(3-aminophenyl)pyrazin-2-amine in ethanol is required. The structural characterization was performed by ^1^H NMR, ^13^C NMR spectroscopy, and FT-MS spectrometry. The results are available in the Supplementary Information.

### 3.2. Photophysical Characterization

The fluorescent spectra of **Clm-1-3** were measured with a Horiba Jovin Fluoromax 4 spectrofluorimeter, with an integration time of 0.1 s. Slit widths of 5 nm were used for both the excitation and emission monochromators. The absorbance spectra were measured using a VWR^®^ Spectrophotometer (UV-3100PC) (VWR, Radnor, PA, USA). Quartz cells with a 10 mm pathlength were used. The spectra were obtained in different solvents for each compound: acetonitrile (CAN, ≥99.9%)), *N*,*N*-dimethylformamide (DMF, ≥99.8%)), ethanol (EtOH, 96%), methanol (MeOH, ≥99.9%), and deionized water (H_2_O). All compounds were analyzed at a final concentration of 30 µM.

### 3.3. In Vitro Studies

#### 3.3.1. Cell Culture

MRC-5 human normal lung fibroblast cell line was used for evaluation of the biosafety profile of the compounds. MCF-7 human mammary carcinoma and PC-3 human prostate carcinoma cell lines were used for evaluation of the anticancer effect of the compounds previously synthesized. These cell lines were obtained from the American Type Culture Collection (ATCC; Manassas, VA, USA) and maintained in Dulbecco’s modified Eagle’s medium (DMEM) cell culture supplemented with 10% fetal bovine serum (FBS) and 1% penicillin–streptomycin (pen–strep) solution, at 37 °C and 5% CO_2._ These reagents were purchased on Millipore Sigma (Merck KGaA, Darmstadt, Germany). For maintenance, confluent cells were trypsinized using a 0.25% trypsin–EDTA solution (Gibco; Thermo Fisher Scientific, Inc., Waltham, MA, USA) and subcultured in the same culture medium. The medium was changed every 96 h. Before treatments, 40,000 (MCR-5), 50,000 (MCF-7), and 25,000 (PC-3) cells/mL were seeded in 96-well plates and allowed to adhere overnight. Cells were used in passages 8, 45, and 15, respectively.

#### 3.3.2. Drug Treatment

The toxicological effect of each compound was evaluated after 48 h of treatment. Cells were treated with each compound in the following concentrations: 1, 25, 50, and 100 µM. Control cells were treated with 0.1% methanol (vehicle). For the evaluation of the toxicological effect of the combination of 5-Fluorouracil (5-FU) and **Clm-1**, MCF-7, and PC-3 cells were treated with the IC_50_ of 5-FU (11.79 µM) and increasing concentrations **Clm-1** (1, 25, 50, and 100 µM), for 48 h. Controls were treated with 0.1% of methanol and 0.1% of DMSO (vehicles).

#### 3.3.3. Morphological Analysis

The toxicological effect was evaluated by morphological analysis and MTT assay. Cell morphology was evaluated after each treatment using a Leica DMI 6000 B microscope equipped with a Leica DFCLM-350 FX camera (Leica Microsystems, Wetzlar, Germany). Images were analyzed with the Leica LAS X imaging software (v3.7.4) (Leica Microsystems, Wetzlar, Germany).

#### 3.3.4. MTT Assay

The biosafety profiles and anticancer effects of each compound were evaluated by an MTT (thiazolyl blue tetrazolium bromide) assay. Briefly, after each treatment, cell media were aspirated and 100 µL of MTT solution (0.5 mg/mL in PBS; cat. no. M5655; Sigma-Aldrich; Merck KGaA, Darmstadt, Germany) was added to each well; plates were incubated for 3 h at 37 °C in the dark. Subsequently, MTT solution was removed from each well and 100 µL of DMSO was added to each well to solubilize the formazan crystals. Absorbance was measured at 570 nm using an automated microplate reader (Tecan Infinite M200, Tecan Group Ltd., Männedorf, Switzerland). Cell viability was calculated as follows:

$$\mathrm{Cell}\;\mathrm{viability}\;\left(\%\right)=\frac{\mathrm{optical}\;\mathrm{density}\;\mathrm{of}\;\mathrm{experimental}\;\mathrm{groups}}{\mathrm{optical}\;\mathrm{density}\;\mathrm{of}\;\mathrm{control}\;\mathrm{group}}\times 100$$

#### 3.3.5. Statistical Analysis

Experiments were conducted in triplicate (*n* = 3). GraphPad Prism 9 (GraphPad Software Inc., San Diego, CA, USA) was used to obtain the cell viability graphs. Results are represented as the mean ± SEM for n experiments performed. Statistical analysis was performed with one-way ANOVA tests by Dunnett’s multiple comparisons between control and treatment groups. Statistical significance was accepted at *p* values < 0.05.

## 4. Conclusions

Here, we investigated the anticancer potential of brominated analogs of coelenteramine, a metabolic product and synthesis precursor of the chemi-/bioluminescent systems of marine coelenterazine. To that end, we designed and synthesized new coelenteramine analogs and evaluated their cytotoxicity (as well as that of previously developed analogs) toward both breast and prostate cancer cell lines. Our objectives were to obtain further insight into the structure–activity relationship of these compounds, and to further clarify whether and how their mechanism of action is related with the family of brominated coelenterazine analogs with anticancer activity (which are structurally very similar).

Interestingly, we found that, despite their structural similarity (imidazopyrazinone core versus an aminopyrazine one), brominated coelenterazines and coelenteramines present quite distinct behaviors and potencies. Thus, these types of compounds should be considered as anticancer molecules with independent activities. We have also obtained evidence that the anticancer activity of brominated coelenteramines is structurally quite inflexible, being quite specific for the 3-(4-bromophenyl)pyrazin-2-amine structure. Finally, we also found that combining the best-performing coelenteramine analog improves the profile of a known chemotherapeutic agent, even at lower concentrations where its anticancer activity by itself is not relevant. Thus, it shows potential to reduce the dose required for chemotherapeutic drugs, while maintaining their efficiency.

In summary, our results show that the chemi-/bioluminescent systems of marine coelenterazine are an attractive and unexpected source of different compounds with distinct and useful anticancer properties. Additionally, the obtained information should be useful in guiding future optimizations of these anticancer compounds.

## Supplementary Materials

The following supporting information can be downloaded at: https://www.mdpi.com/article/10.3390/ijms232213981/s1. General Synthetic Procedures for **Clm 1-3** and 5-(3-Aminophenyl)pyrazin-2-amine. 2D excitation-emission matrices (EEMs) of **Clm-1** (Figure S1), **Clm-2** (Figure S2) and **Clm-3** (Figure S3). ^1^H-NMR, ^13^C-NMR, COSY-NMR, HMBC-NMR, HSQC-NMR spectra and FTMS-ESI (+) spectrum for **Clm-2** (Figures S4–S9, respectively), **Clm-3** (Figures S13–S18, respectively), ^1^H-NMR, ^13^C-NMR, and FTMS-ESI (+) spectrum for 5-(3-Aminophenyl)pyrazin-2-amine (Figures S10–S12, respectively). Morphological evaluation of MCF-7 (Figure S19), PC-3 (Figure S20) and MRC-5 (Figure S21) cells treated with **Clm-1-3**. Morphological evaluation of MCF-7 (Figure S22) and PC-3 (Figure S23) cells treated with a combination of 5-FU (11.79 µM) and different concentrations of **Clm-1**.
