# Supplementary material for: Investigation of the Anticancer and Drug Combination Potential of Brominated Coelenteramines toward Breast and Prostate Cancer
Source: Int J Mol Sci. 2022 Nov 12;23(22):13981. doi: 10.3390/ijms232213981 (PMC9699044; doi:10.3390/ijms232213981)
Supplement: Supplementary file 1 [file ijms-23-13981-s001.zip › ijms-1971906-supplementary.pdf]

Table of Contents

1. Experimental section 2
1.1. General Procedures 2
1.2. Instruments and data analysis 3
1.2.1. NMR 3
1.2.2. FT-MS 3
1.2.3. ESI-MS 3
1.2.4. UV-Vis Spectra 3
1.3. Photophysical Characterization 3
1.4. Synthesis and characterization 3
1.4.1. 3,5-Bis(4-bromophenyl)pyrazin-2-amine 3
1.4.2. 5-(3-Aminophenyl)pyrazin-2-amine 4
1.4.1. 5-(5-Amino-2,4-dibromophenyl)pyrazin-2-amine 5
2. Supporting Figures 6
2.1. Photophysical characterization 6
2.2. 3,5-Bis(4-bromophenyl)pyrazin-2-amine 7
2.3. 5-(3-Aminophenyl)pyrazin-2-amine 11
2.4. 5-(5-Amino-2,4-dibromophenyl)pyrazin-2-amine 14
2.5. Morphological Analysis 18

1. Experimental section

1.1. General Procedures

Reagents and solvents were purchased from Millipore Sigma (Merck KGaA, Darmstadt, Germany) and used without further purification. All reactions involving oxygen or moisture-sensitive compounds were carried out under dry nitrogen atmosphere. Ice-water and silicon baths were used for reactions at low and high temperatures, respectively, with all reaction temperatures referring to the external bath. Organic extracts were dried over anhydrous Na_2SO_4, filtered and concentrated using a rotary evaporator (Büchi® Rotavapor® R-210, Büchi® B-491 Heating Bath 120V, KNF Neuberger D-79112 Vacuum Pump N 035.1.2 AN.18).

Reactions were monitored by thin-layer chromatography (TLC) using aluminum-backed Merck 60 F_{254} silica gel plates and *n*-hexanes-ethyl acetate solvent systems. After visualization under ultraviolet light at 254 nm and 365 nm, the plates were developed by immersion in a solution containing a mixture of *p*-anisaldehyde (2.5%), acetic acid (1%), and sulfuric acid (3.4%) in 95% ethanol followed by heating. Solid compounds were mixed with SiO_2, redissolved in CH_2Cl_2, and concentrated under reduced pressure before purification through column chromatography using silica gel (Aldrich, 230-400 mesh) and EtOAc-hexanes mixtures. Compounds were systematically named following IUPAC recommendations with ChemDraw 20.0.0.41.

Cell lines were obtained from the American Type Culture Collection (ATCC; Manassas, VA, USA). Dulbecco's modified Eagle's medium (DMEM) cell culture medium and other cell reagents were purchased on Millipore Sigma.

Abbreviations: ACN = Acetonitrile, bs = Broad singlet; DMF = Dimethylformamide, DMSO = Dimethyl sulfoxide; d =doublet; EtOAc = Ethyl acetate; EtOH = Ethanol; FTMS = Fourier transform mass spectrometry; hep = heptet; HSQC = Heteronuclear Single Quantum Coherence; HMBC = Heteronuclear Multiple Bond Correlation; MeOH = Methanol; NBS = N-Bromosuccinimide; NMR = Nuclear Magentic Resonance; THF = Tetrahydrofuran; TLC: Thin Layer Chromatography; t = triplet.

1.2. Instruments and data analysis

1.2.1. NMR

NMR spectra were recorded in $CDCl_3$ or Acetone-d6. solutions on a Bruker NMR spectrometer (Bruker Advance III 400 MHz Ascend, 9.4 Tesla), and chemical shifts are reported on the δ scale (ppm) using the residual solvent signals [δ = 7.26 ppm (^1H, $CDCl_3$), δ = 77.0 ppm (^{13}C, t, $CDCl_3$); δ = 4.87 ppm (^1H, Methanol-d_4), δ = 49.0 ppm (^{13}C, hep, Methanol-d_4)] as internal standards. Coupling constants (*J*) are reported in Hz.

1.2.2. FT-MS

FT-MS analysis was done on a LTQ OrbitrapTM XL hybrid mass spectrometer (Thermo Fischer Scientific, Bremen, Germany) controlled by LTQ Tune Plus and Xcalibur 2.1.0.

1.2.3. ESI-MS

Direct injection ESI-MS was made using a Thermo Finnigan™ LCQ™ Deca XP Max (Thermo Electron Corporation, Waltham, USA) mass spectrometer. It was operated as follows: spray voltage, 5 kV; capillary voltage, ±15 V; capillary temperature, 300 ºC.

1.2.4. UV-Vis Spectra

The absorbance spectra were measured using a VWR® Spectrophotometer (UV-3100PC), and a quartz cuvette Micro Cell 115F-QS LP with a 10x2mm light path (Hellma Analytics). Solvents were purchased from Merck and were HPLC-grade.

1.3. Photophysical Characterization

Stock solutions of the compounds were prepared by dissolving 2 mg of the compounds in 5 mL of MeOH, and then stored at -80º C until use. 30µM solutions were prepared for each compound from stock solution.

The fluorescent and absorbance spectra of the reaction mixtures were performed in ACN, DMF, EtOH, H_2O and MeOH.

1.4. Synthesis and characterization

1.4.1. 3,5-Bis(4-bromophenyl)pyrazin-2-amine

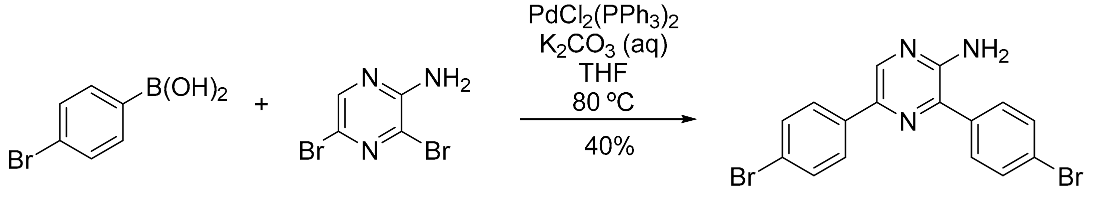

An aqueous solution of K_2CO_3 (1M, 9.25 mmol, 7.5 eq) was added to a stirring solution of (4-bromophenyl)boronic acid (0.496 g, 2.469 mmol, 2 eq) and 3,5-dibromopyrazin-2-amine (0.312 g, 1.234 mmol, 1 eq) in THF (9 mL) and was deoxygenated with N_2. Then $PdCl_2(PPh_3)_2$ (0.043 g, 0.075 mmol, 0.05 eq) was added and the resulting mixture was stirred at 80 ºC until no starting material was detected by TLC (1:1 EtOAc-hexanes). The reaction mixture was cooled to *rt* and the aqueous phase discharged. The combined organic layers were washed with brine, dried with anhydrous sodium sulfate, filtered, and concentrated under reduced pressure to give an orange solid, which was purified by column chromatography (SiO_2, EtOAc/hexanes gradient) to afford 3,5-bis(4-bromophenyl)pyrazin-2-amine as a yellow solid [0.200 g, 40%, R_f = 0.64 (10% EtOAc/hex)].

^{1}H NMR (400 MHz, $CDCl_3$) δ = 8.42 (s, 1H), 7.83 (d, *J* = 8.7 Hz, 2H), 7.71 (d, *J* = 8.7 Hz, 2H), 7.67 (d, *J* = 8.7 Hz, 2H), 7.57 (d, *J* = 8.7 Hz, 2H), 4.95 (bs, 2H).

^{13}C NMR (101 MHz, $CDCl_3$) δ = 151 (C), 142.2 (C), 137.8 (CH), 132.4 (CH), 132.1 (CH), 130.1 (CH), 127.4 (CH), 123.7 (C), 122.7 (C).

1.4.2. 5-(3-Aminophenyl)pyrazin-2-amine

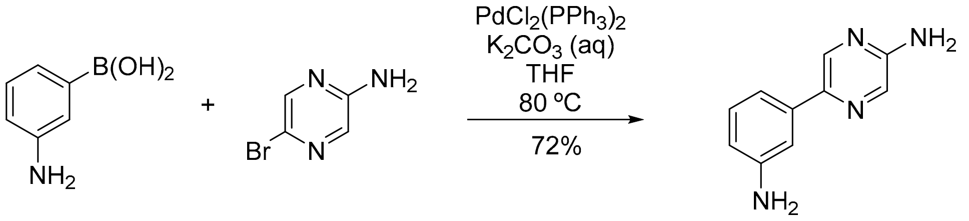

An aqueous solution of K_2CO_3 (1M, 18.64 mmol, 7.5 eq) was added to a stirring solution of (3-aminophenyl)boronic acid (0.462 g, 2.982 mmol, 1.2 eq) and 5-bromopyrazin-2-amine (0.432 g, 2.485 mmol, 1 eq) in THF (20 mL) and was deoxygenated with bubbling Argon. Then $PdCl_2(PPh_3)_2$ (0.174 g, 0.249 mmol, 0.1 eq) was added and the resulting mixture was stirred at 80 ºC until no starting material was detected by TLC (1:1 EtOAc-hexanes). The reaction mixture was cooled to room temperature and the aqueous phase discharged. The combined organic layers were washed with brine, dried with anhydrous sodium sulfate, filtered, and concentrated under reduced pressure to give an orange solid, which was purified by column chromatography (SiO_2, EtOAc/hexanes gradient) to afford 5-(3-aminophenyl)pyrazin-2-amine as an yellow foam [0.410 g, 72%, R_f = 0.24 (30% EtOAc/hex)].

^{1}H NMR (400 MHz, $(CD_3)_2CO$) δ = 8.39 (d, *J* = 1.6 Hz, 1H), 8.03 (d, *J* = 1.5 Hz, 1H), 7.30-7.29 (m, 1H), 7.19- 7.16 (m, 1H), 7.10 (t, *J* = 7.7 Hz, 1H), 6.64 (ddd, *J* = 7.8, 2.3, 1.1 Hz, 1H), 5.82 (bs, 2H), 4.65 (bs, 2H).

^{13}C NMR (101 MHz, Acetone-d6) δ = 155.7 (C), 149.7 (C), 142.4 (C), 139.5 (CH), 132.2 (CH), 130.1 (CH), 114.7 (CH), 112.1 (CH).

FTMS-ESI (+): m/z: calcd for $[C_{10}H_{10}N_4]^+$: 186.0905 $[M+H]^+$; found 187.1008 $[C_{10}H_{10}{}^{14}N_4]^+$, 188.1043 $[C_{10}H_{10}{}^{15}N_4]^+$.

1.4.1. 5-(5-Amino-2,4-dibromophenyl)pyrazin-2-amine

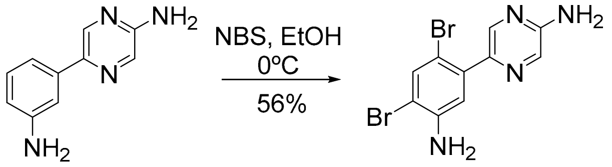

NBS (0.765 g, 4.296 mmol, 2 eq) was added to a solution of 5-(3-aminophenyl)pyrazin-2-amine (0.400 g, 2.148 mmol, 1 eq) in ethanol (30 mL), which was previously cooled to 0 ºC, and stirred at that temperature for 5 min. The reaction mixture was then diluted with EtOAc and washed with brine. The combined organic layers were dried with anhydrous sodium sulfate, filtered, and concentrated under reduced pressure to give a dark orange solid, which was purified by column chromatography (SiO_2, hexanes-EtOAc gradient) to afford 5-(5-amino-2,4-dibromophenyl)pyrazin-2-amine as a light yellow solid [0.333 g, 72 %, R_f = 0.68 (50% EtOAc/hex)].

^{1}H NMR (400 MHz, $CDCl_3$) δ = 8.06 (s, 1H), 7.94 (s, 1H), 7.33 (dd, *J* = 8.6, 1.1 Hz, 1H), 6.66 (dd, *J* = 8.6, 1.0 Hz, 1H), 5.09 (bs, 2H), 4.20 (bs, 1H).

^{13}C NMR (101 MHz, $CDCl_3$) δ = 178.4 (C), 153.2 (C), 144.0 (C), 141.9 (CH), 138.5 (C), 131.5 (CH), 131.2 (CH), 116.4 (C), 111.4 (C), 111.3 (C).

2. Supporting Figures

2.1. Photophysical characterization

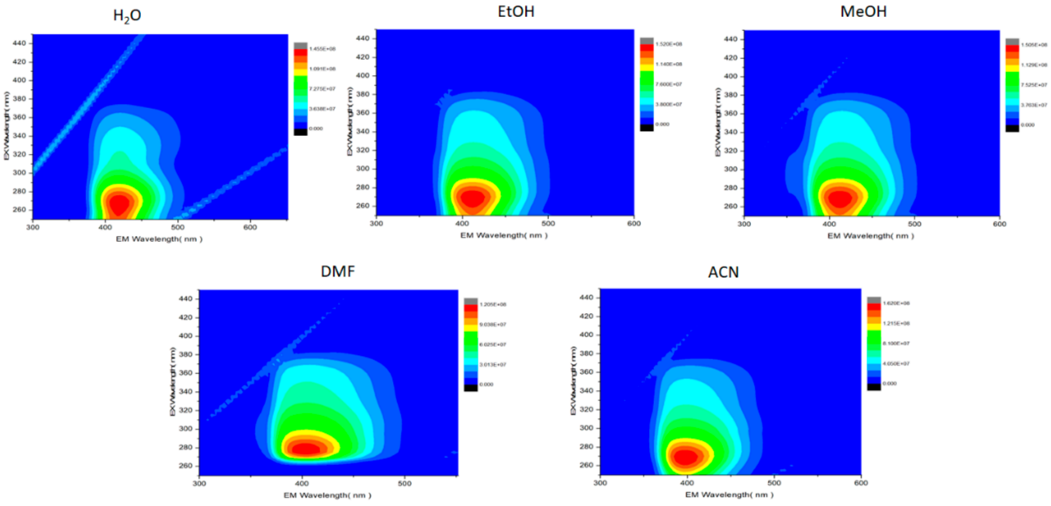

Figure S1. 2D excitation-emission matrices (EEMs) of **Clm-1**, in studied solvents.

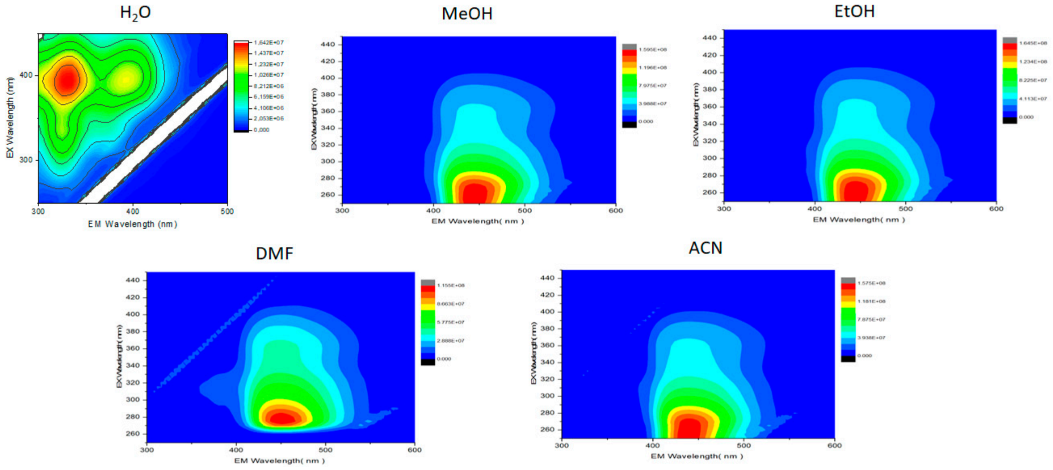

Figure S2. 2D excitation-emission matrices (EEMs) of **Clm-2**, in studied solvents.

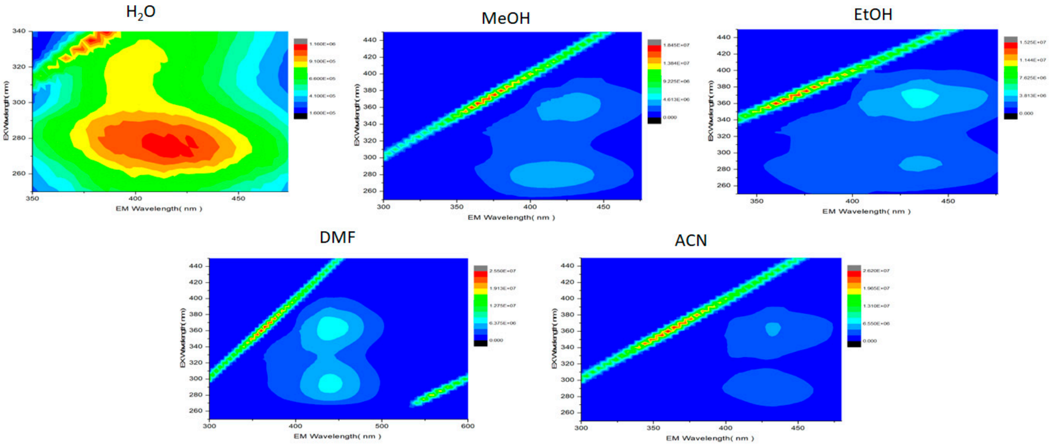

Figure S3. 2D excitation-emission matrices (EEMs) of **Clm-3**, in studied solvents.

2.2. 3,5-Bis(4-bromophenyl)pyrazin-2-amine

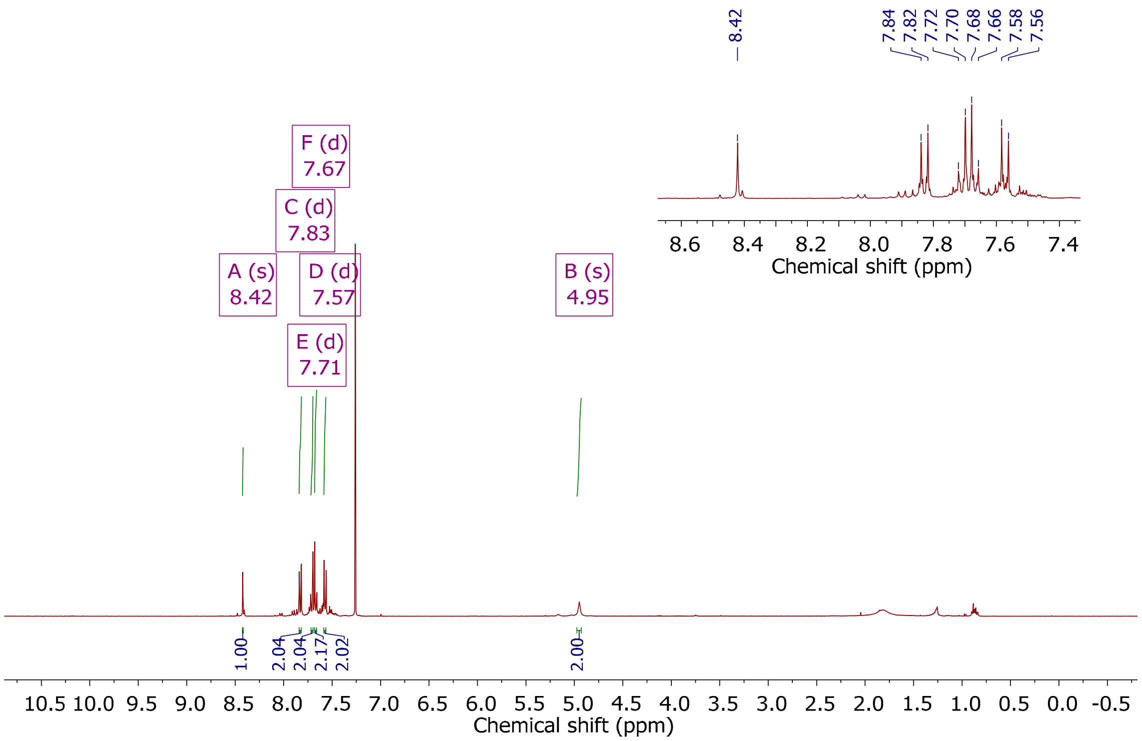

Figure S4. ^{1}H-NMR spectrum of 3,5-bis(4-bromophenyl)pyrazin-2-amine (Clm-2) in $CDCl_3$. **^{1}H NMR** (400 MHz, $CDCl_3$) δ = 8.42 (s, 1H), 7.83 (d, J = 8.7 Hz, 2H), 7.71 (d, J = 8.7 Hz, 2H), 7.67 (d, J = 8.7 Hz, 2H), 7.57 (d, J = 8.7 Hz, 2H), 4.95 (bs, 2H).

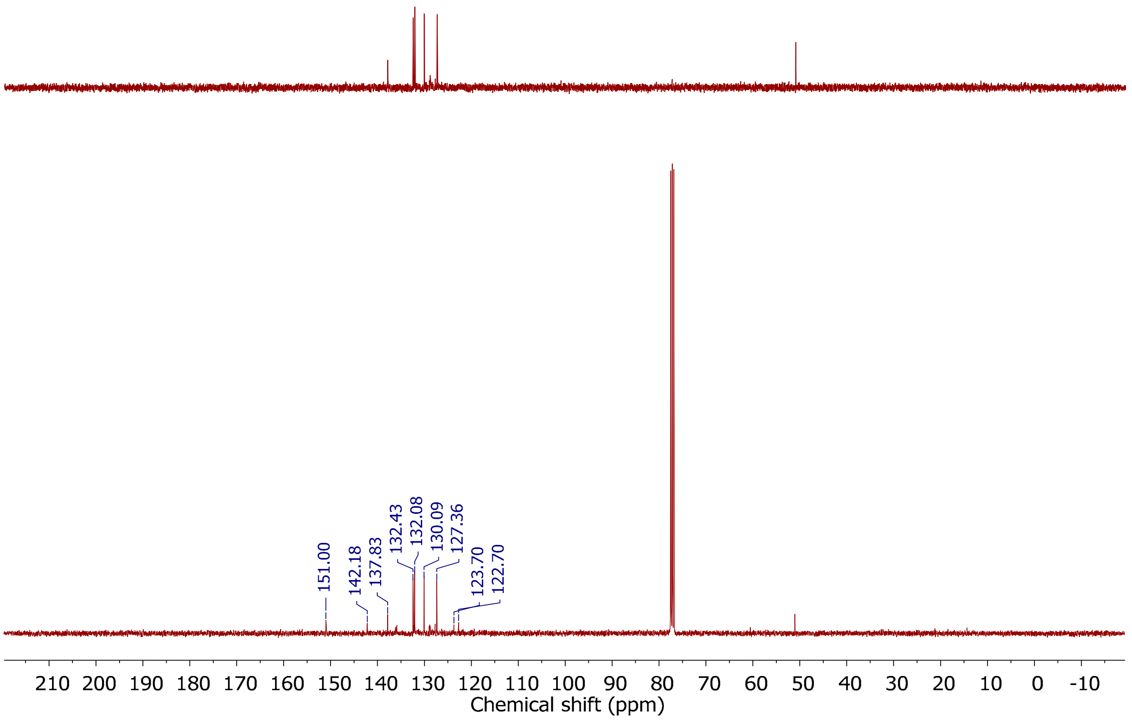

Figure S5. ^{13}C-NMR spectrum of 3,5-bis(4-bromophenyl)pyrazin-2-amine in $CDCl_3$. ^{13}**C NMR** (101 MHz, $CDCl_3$) δ = 151 (C), 142.2 (C), 137.8 (CH), 132.4 (CH), 132.1 (CH), 130.1 (CH), 127.4 (CH), 123.7 (C), 122.7 (C).

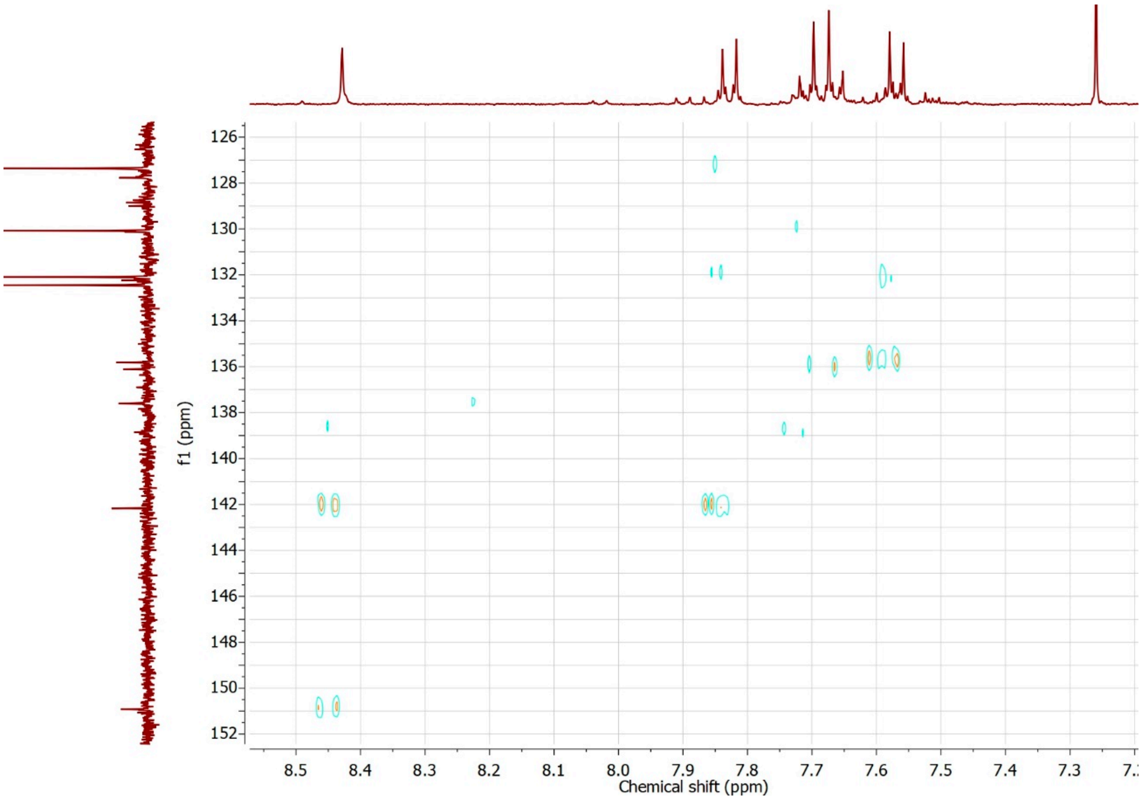

Figure S6. COSY-NMR Spectrum of 3,5-bis(4-bromophenyl)pyrazin-2-amine in $CDCl_3$.

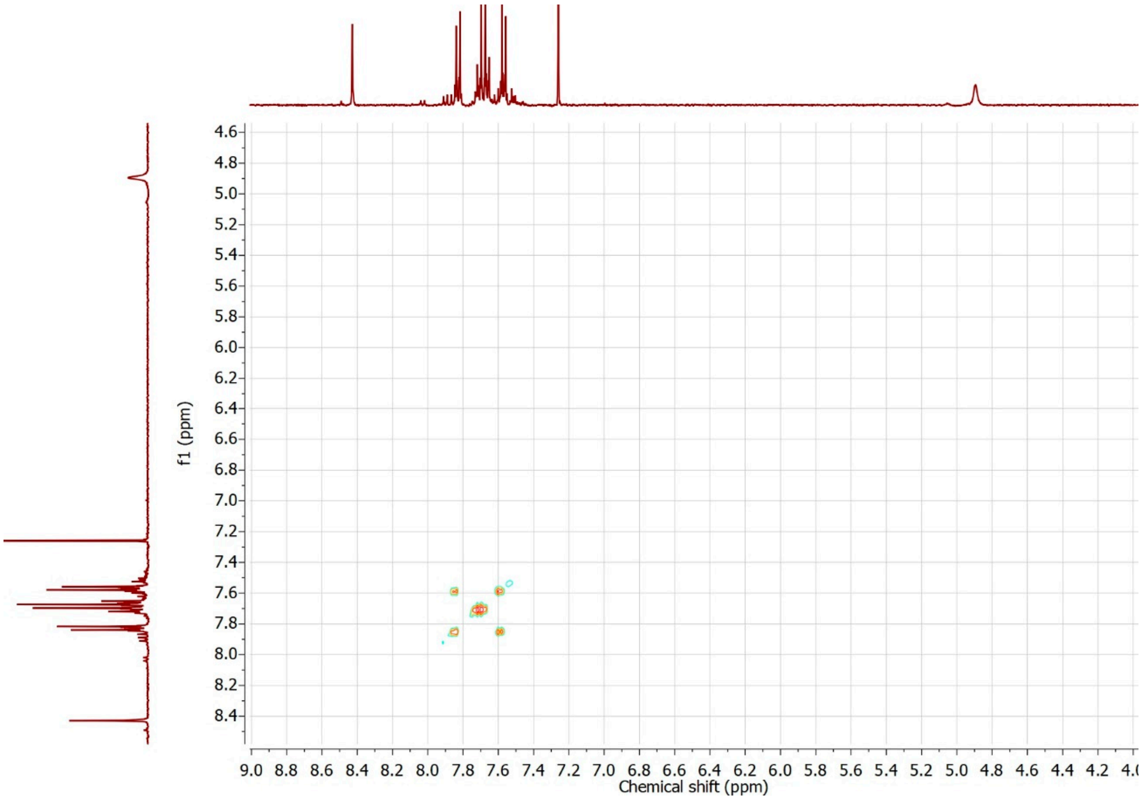

Figure S7. HMBC-NMR spectrum of 3,5-bis(4-bromophenyl)pyrazin-2-amine in $CDCl_3$.

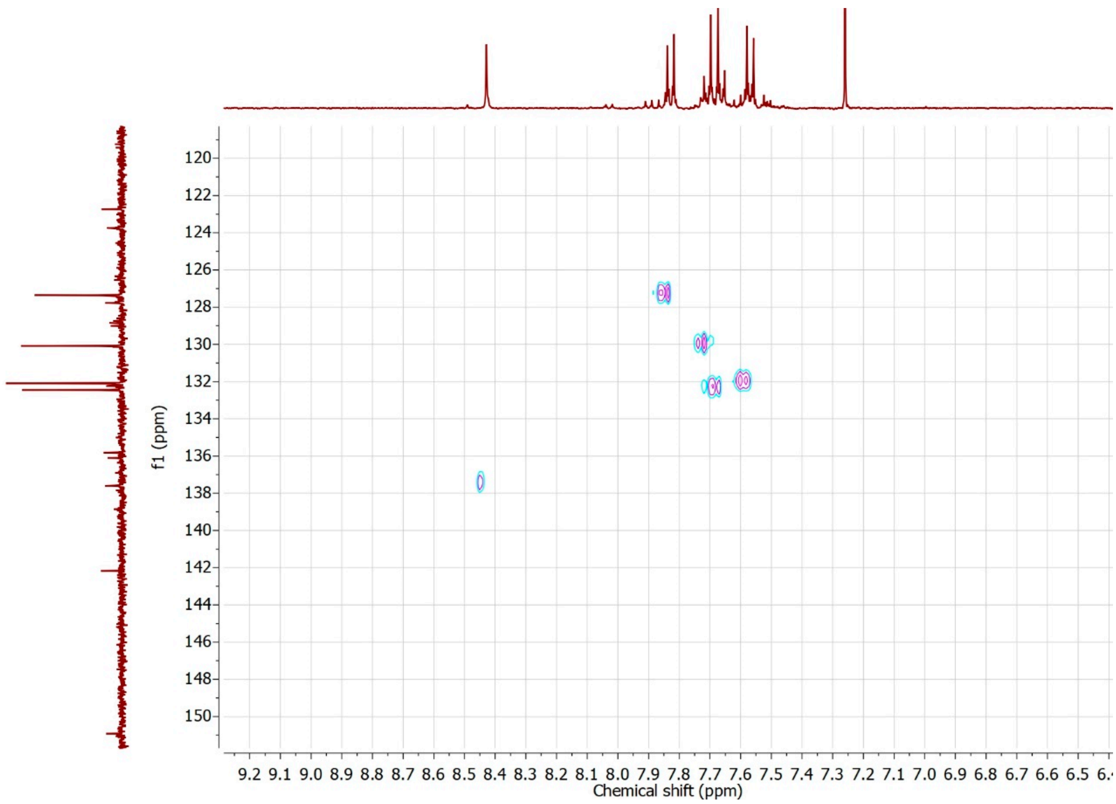

Figure S8. HSQC-NMR spectrum of 3,5-bis(4-bromophenyl)pyrazin-2-amine in $CDCl_3$.

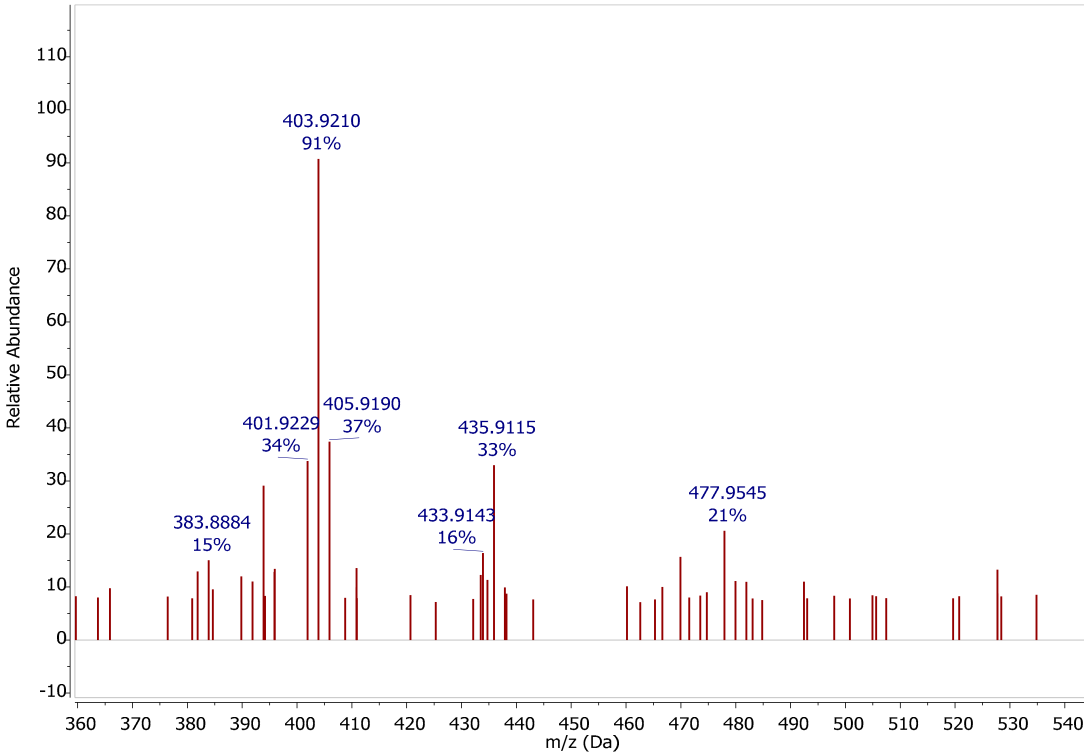

Figure S9. FTMS-ESI (+) spectrum of 3,5-bis(4-bromophenyl)pyrazin-2-amine. FTMS-ESI (+): m/z: calcd for $[C_{16}H_{11}Br_2N_3]^+$: 402.9320 m/z: found 403.9210 $[C_{16}H_{11}{}^{79}Br_2N_3]^+$, 405.9190 $[C_{16}H_{11}{}^{79}Br\,{}^{81}BrN_3]^+$, 401.9229 $[C_{16}H_{11}{}^{81}Br_2N_3]^+$.

2.3. 5-(3-Aminophenyl)pyrazin-2-amine

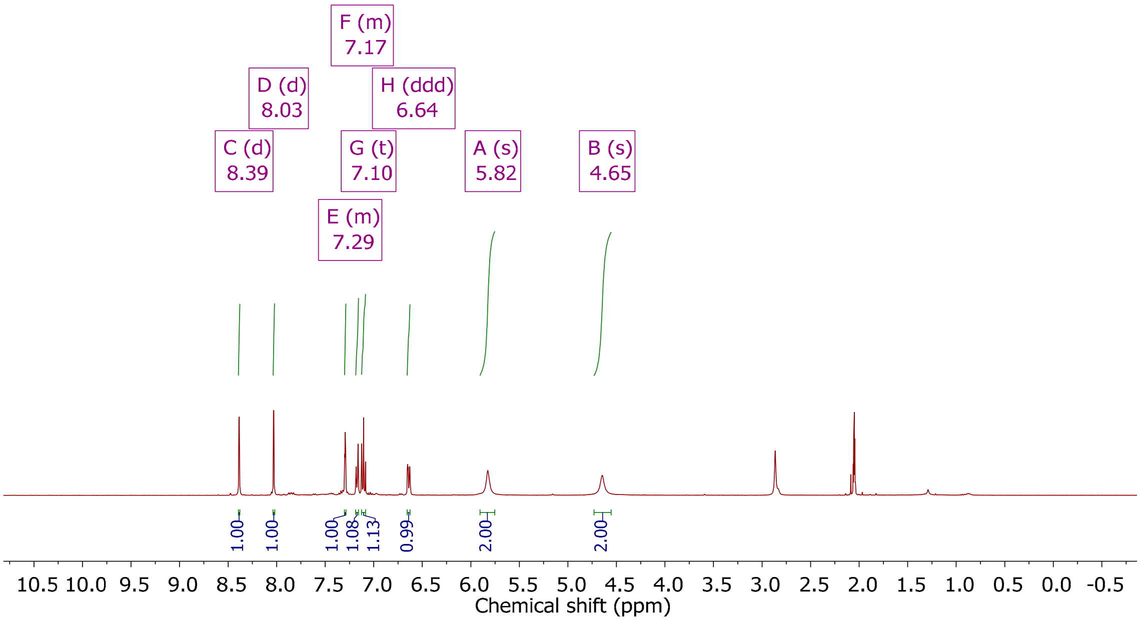

Figure S10. ^{1}H-NMR spectrum of 5-(3-aminophenyl)pyrazin-2-amine in Acetone-d6. **^{1}H NMR** (400 MHz, Acetone-d6) δ = 8.39 (d, *J* = 1.6 Hz, 1H), 8.03 (d, *J* = 1.5 Hz, 1H), 7.30-7.29 (m, 1H), 7.19- 7.16 (m, 1H), 7.10 (t, *J* = 7.7 Hz, 1H), 6.64 (ddd, *J* = 7.8, 2.3, 1.1 Hz, 1H), 5.82 (bs, 2H), 4.65 (bs, 2H).

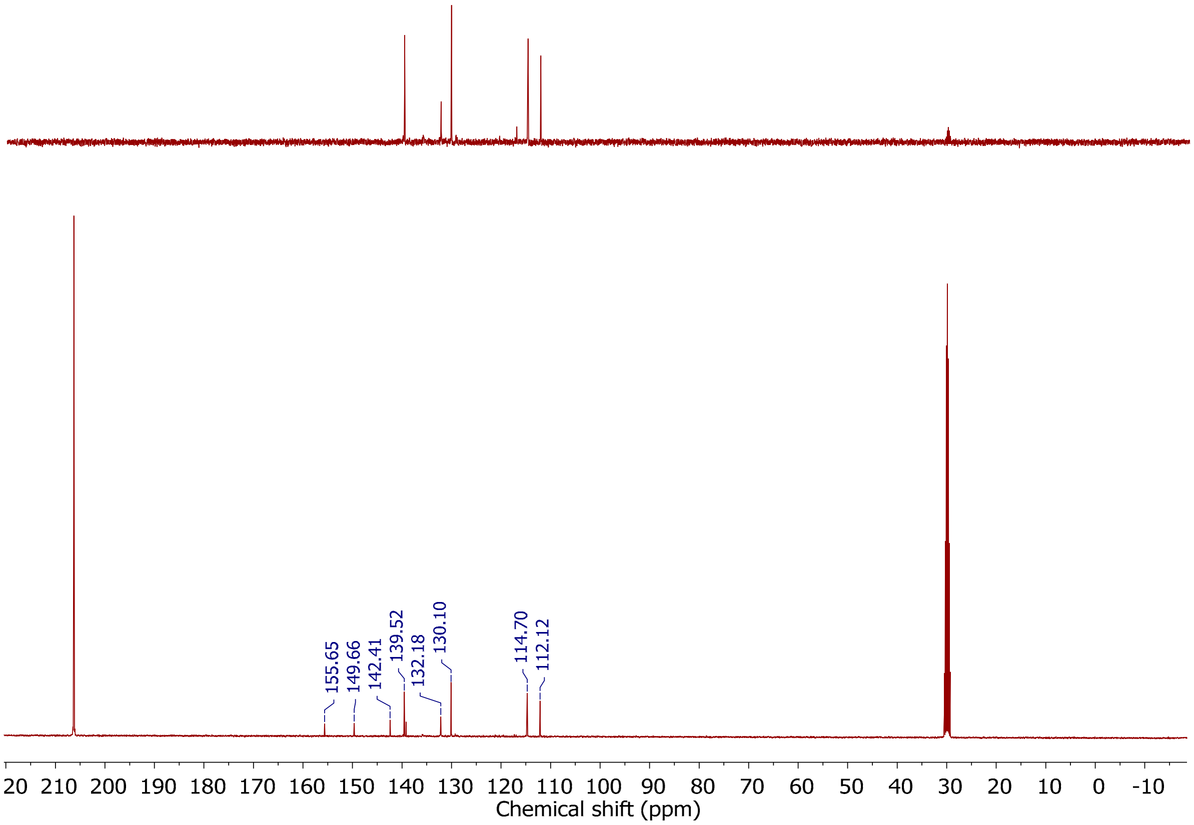

Figure S11. ^{13}C-NMR spectrum of 5-(3-aminophenyl)pyrazin-2-amine in Acetone-d6. **^{13}C NMR** (101 MHz, Acetone-d6) δ = 155.7 (C), 149.7 (C), 142.4 (C), 139.5 (CH), 132.2 (CH), 130.1 (CH), 114.7 (CH), 112.1 (CH).

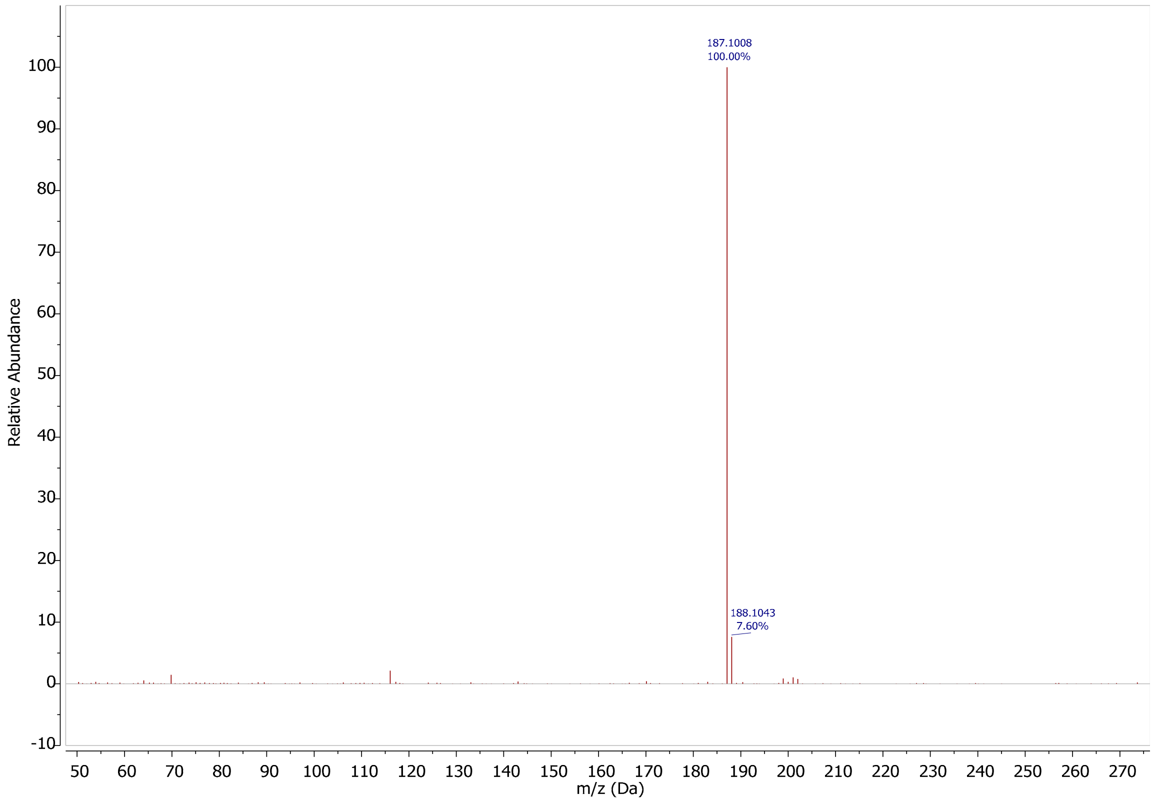

Figure S12. FTMS-ESI (+) spectrum 5-(3-aminophenyl)pyrazin-2-amine. **FTMS-ESI (+):** m/z: calcd for $[C_{10}H_{10}N_4]^+$: 186.0905 $[M+H]^+$; found 187.1008 $[C_{10}H_{10}{}^{14}N_4]^+$, 188.1043 $[C_{10}H_{10}{}^{15}N_4]^+$.

2.4. 5-(5-Amino-2,4-dibromophenyl)pyrazin-2-amine

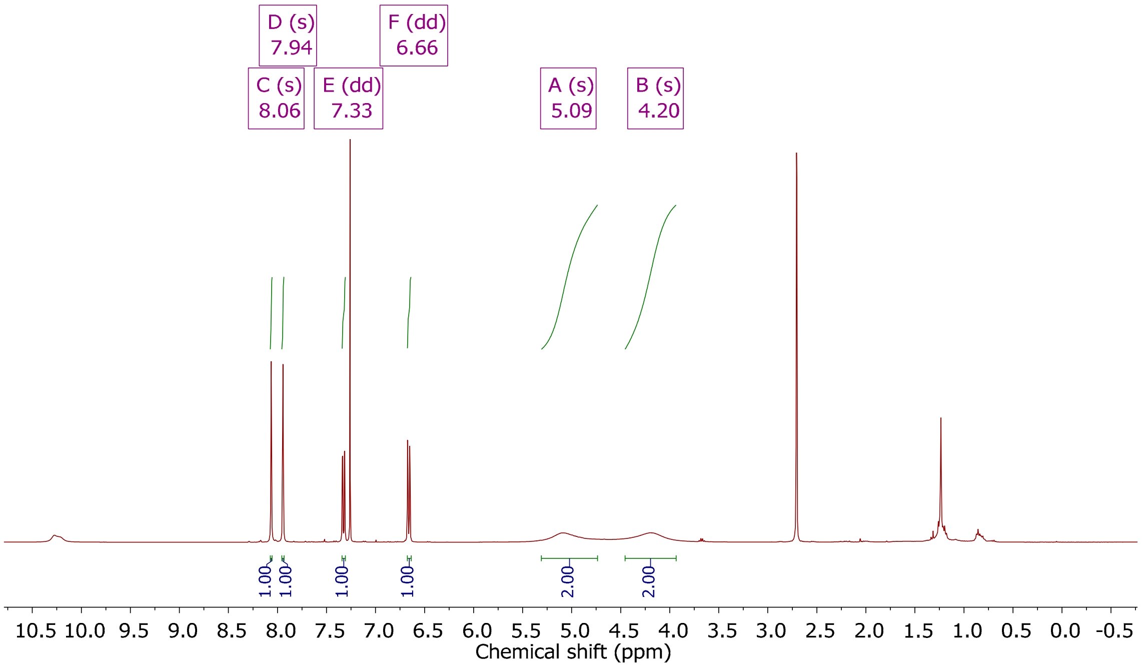

Figure S13. ^{1}H-NMR spectrum of 5-(5-amino-2,4-dibromophenyl)pyrazin-2-amine in $CDCl_3$. **^{1}H NMR** (400 MHz, $CDCl_3$) δ = 8.06 (s, 1H), 7.94 (s, 1H), 7.33 (dd, J = 8.6, 1.1 Hz, 1H), 6.66 (dd, J = 8.6, 1.0 Hz, 1H), 5.09 (bs, 2H), 4.20 (bs, 2).

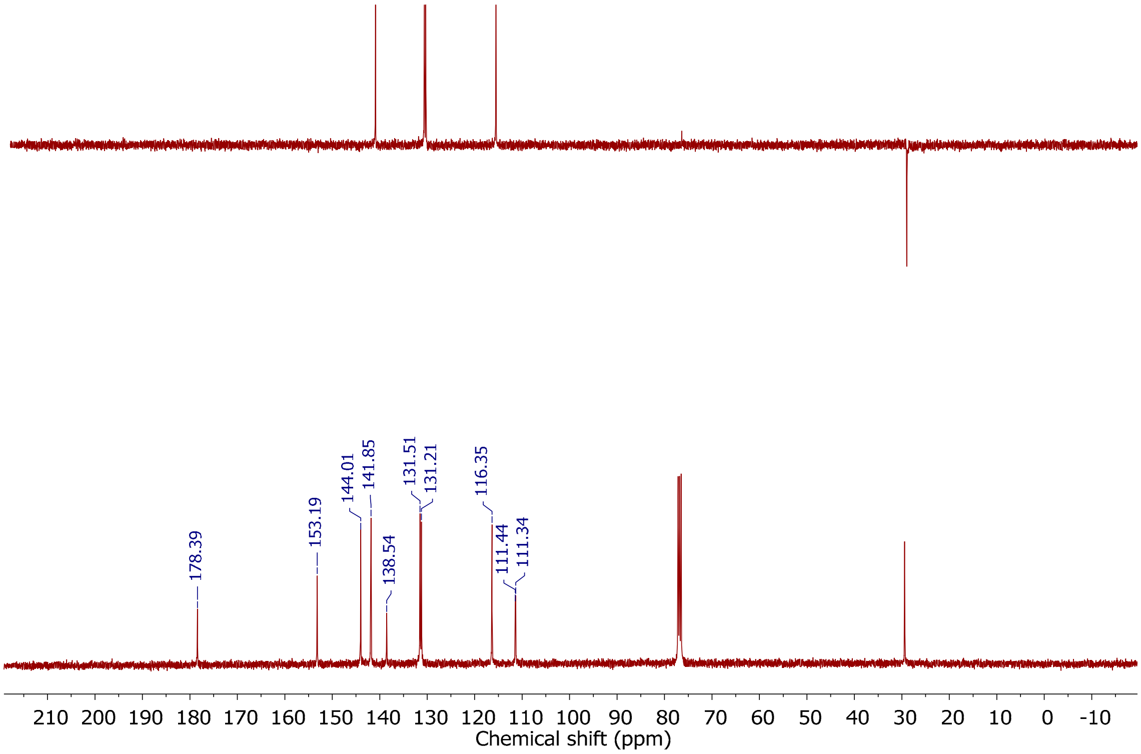

Figure S14. ^{13}C-NMR spectrum of 5-(5-amino-2,4-dibromophenyl)pyrazin-2-amine in ($CDCl_3$). **^{13}C NMR** (101 MHz, $CDCl_3$) δ = 178.4 (C), 153.2 (C), 144.0 (C), 141.9 (CH), 138.5 (C), 131.5 (CH), 131.2 (CH), 116.4 (CH), 111.4 (C), 111.3 (C).

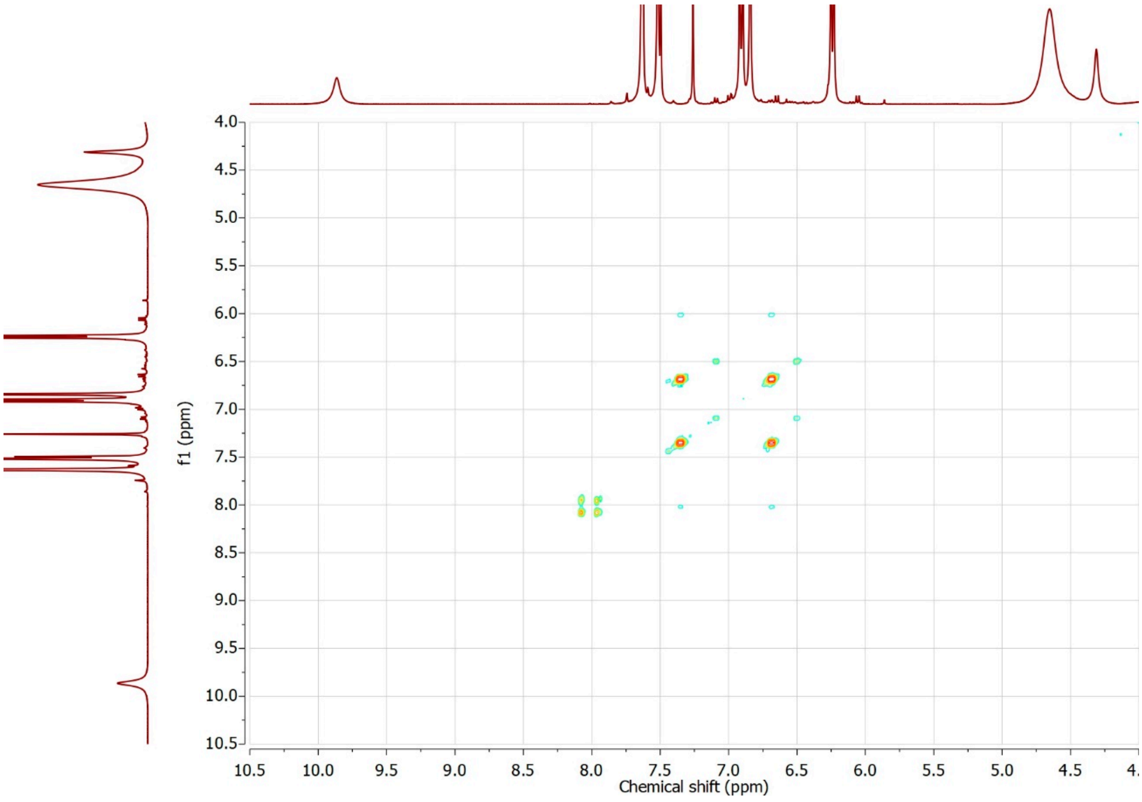

Figure S15. COSY-NMR spectrum of 5-(3-aminophenyl)pyrazin-2-amine in $CDCl_3$.

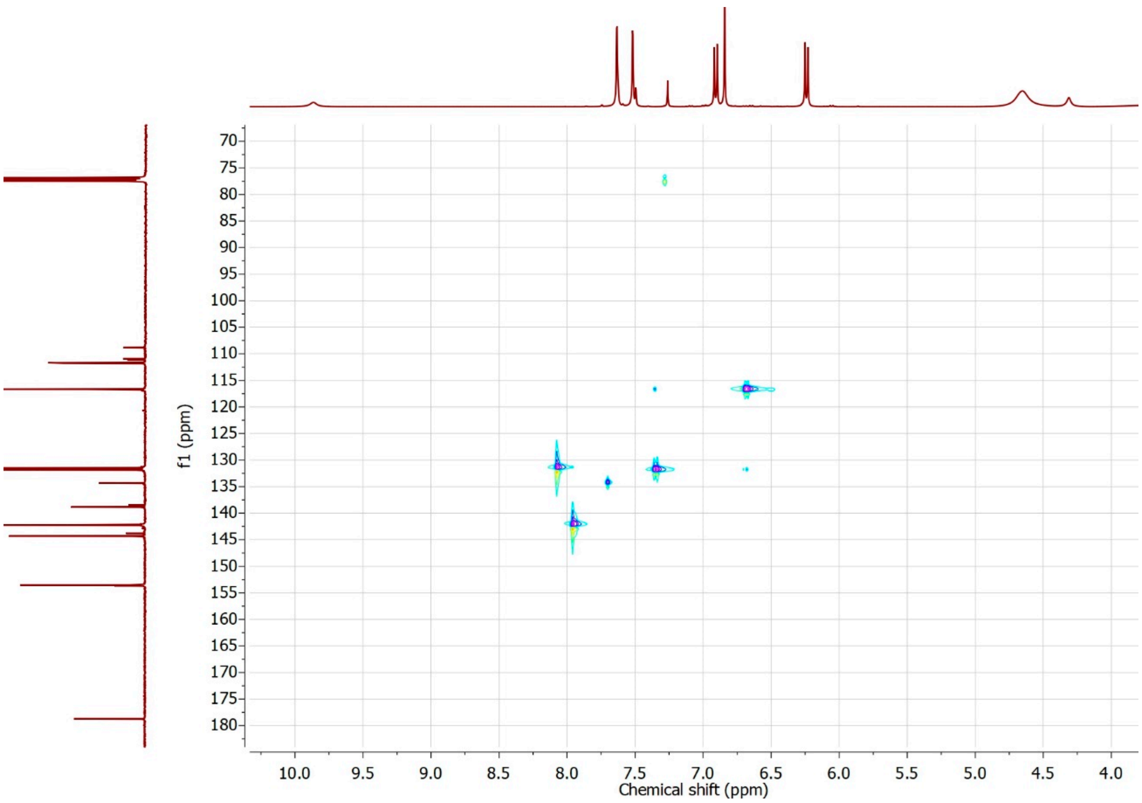

Figure S16. HMBC-NMR spectrum of 5-(3-aminophenyl)pyrazin-2-amine in $CDCl_3$.

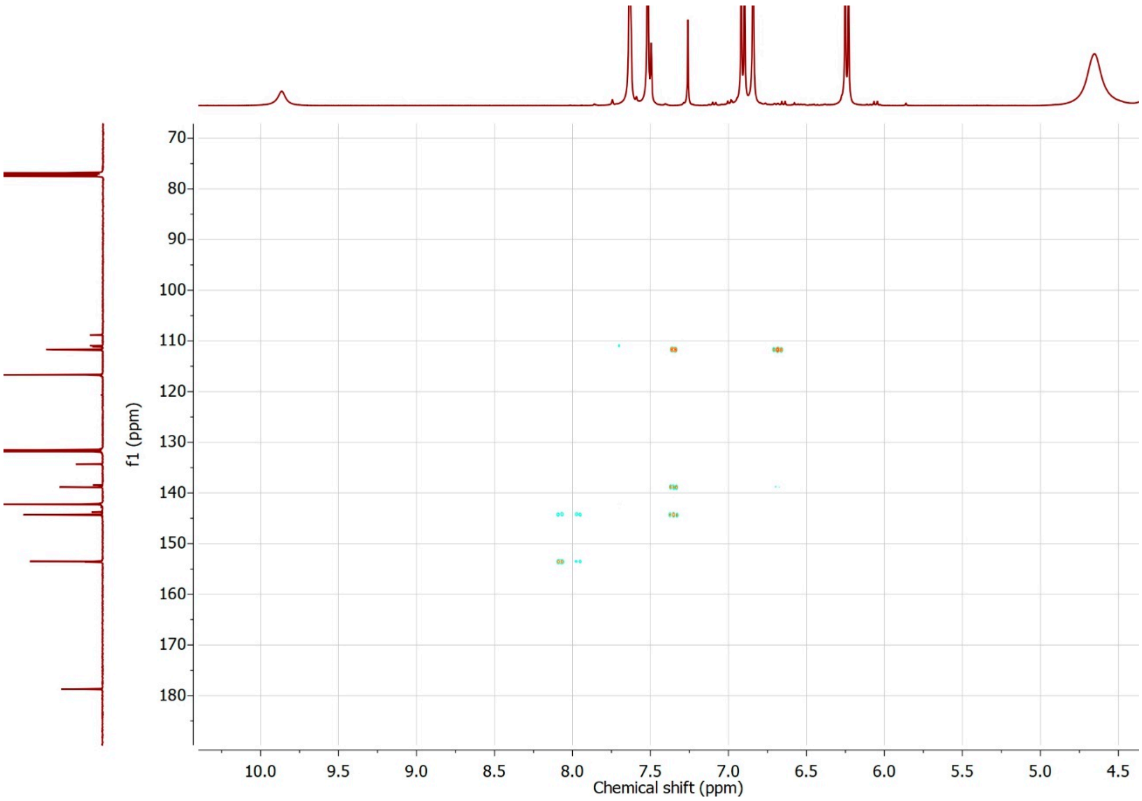

Figure S17. HSQC-NMR spectrum of 5-(3-aminophenyl)pyrazin-2-amine in $CDCl_3$.

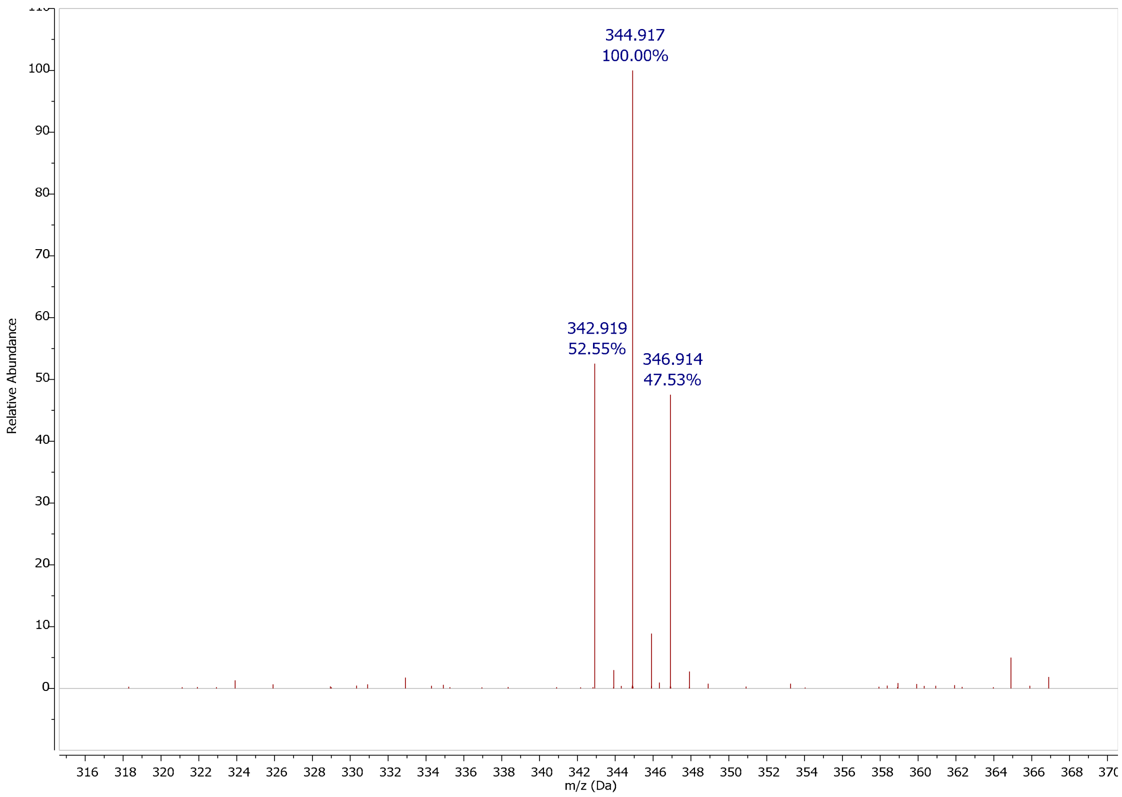

Figure S18. FTMS-ESI (+) spectrum of 5-(5-amino-2,4-dibromophenyl)pyrazin-2-amine. FTMS-ESI (+): m/z: calcd for $[C_{10}H_8Br_2N_4]^+$: 343.9095 m/z: found 344.917 $[C_{10}H_8{}^{79}Br_2N_4]^+$, 342.919 $[C_{10}H_8{}^{79}Br^{81}Br\ N_4]$+, 346.914 $[C_{10}H_8{}^{81}Br_2\ N_4]^+$.

2.5. Morphological Analysis

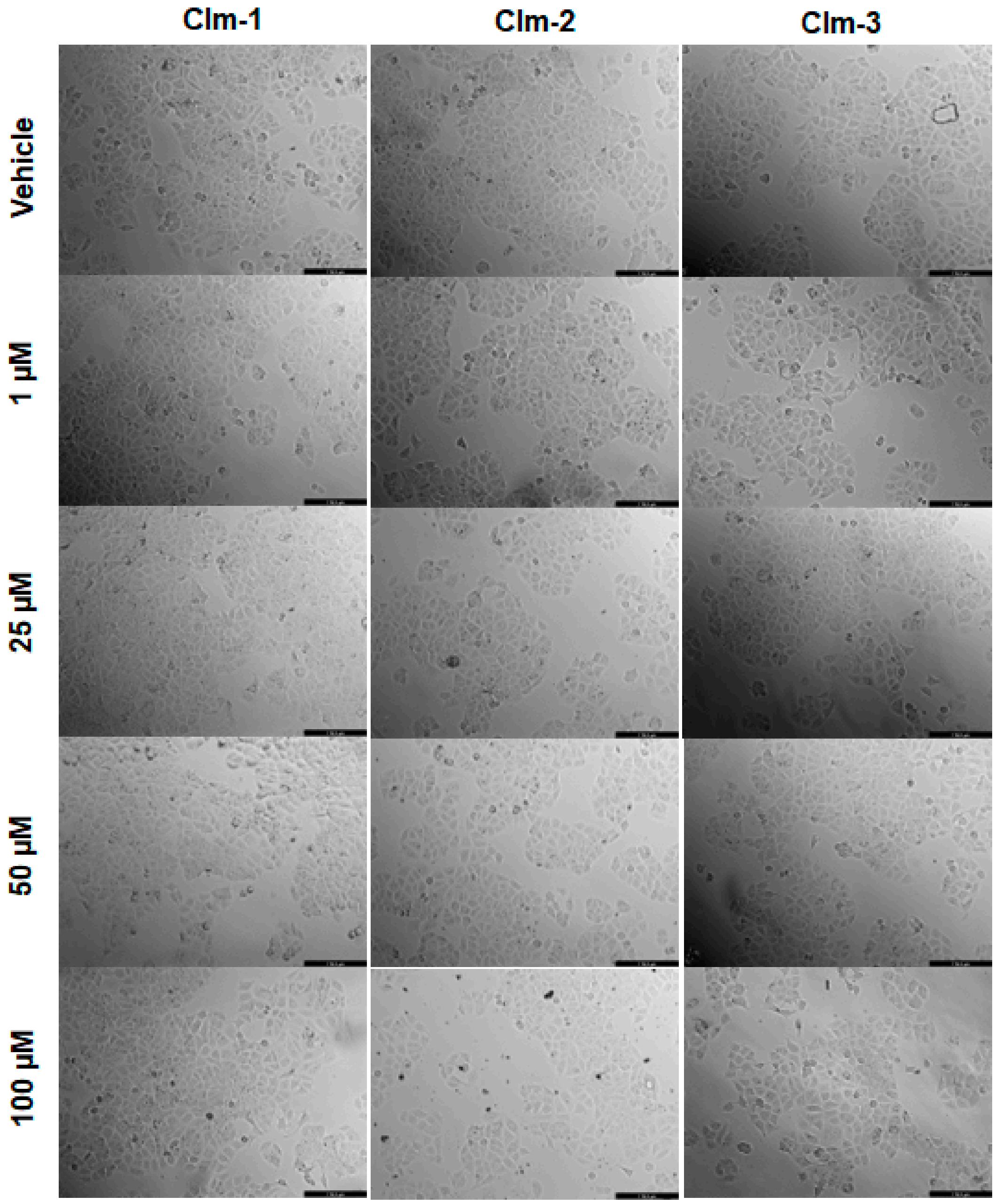

Figure S19. Morphological evaluation of MCF-7 cells treated with each compound. MCF-7 were treated with vehicle (methanol) and increasing concentrations (0-100 µM) of **Clm-1**, **Clm-2** and **Clm-3** for 48 h. Results are representative of three independent experiments. Scale bar: 200 µm.

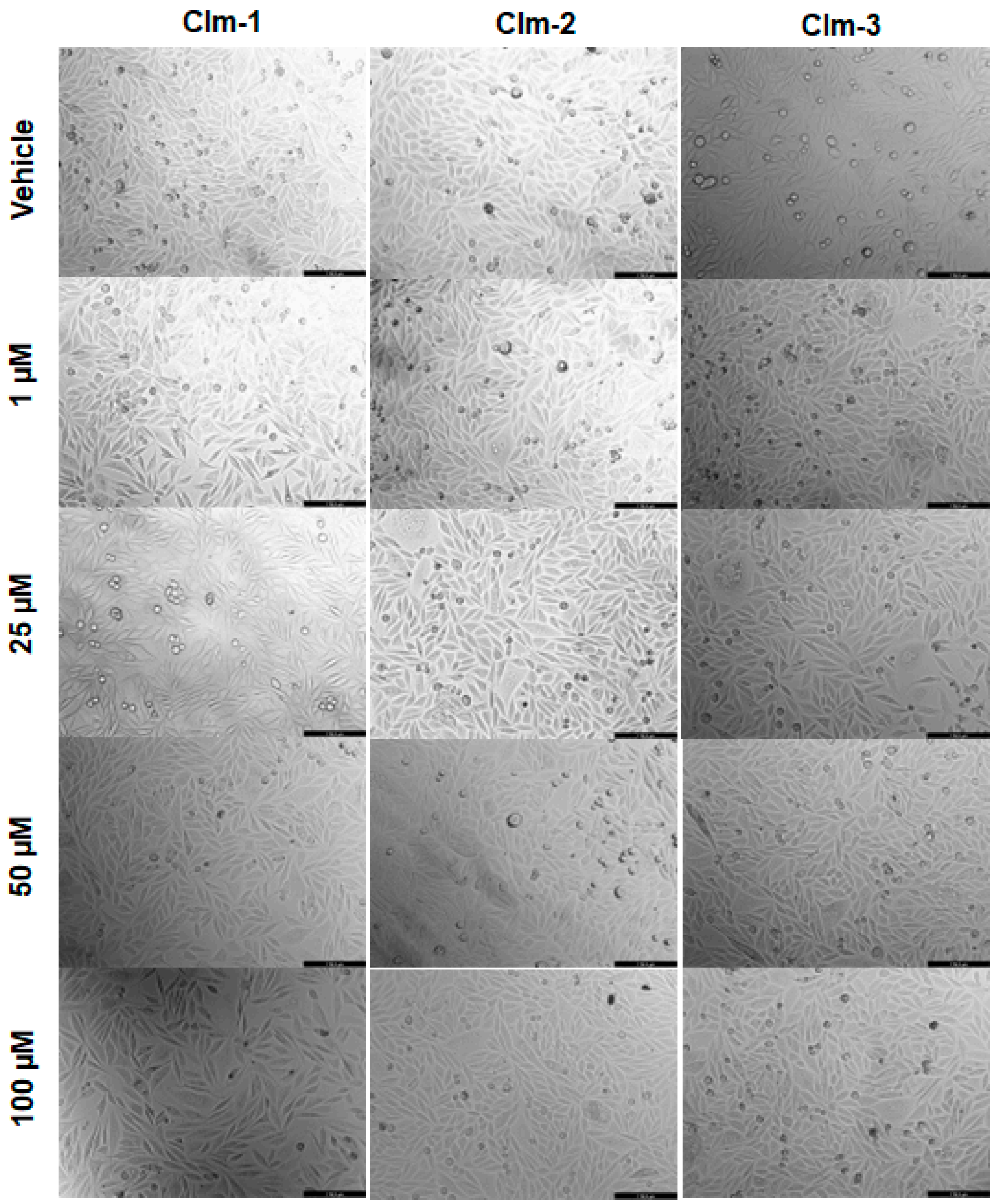

Figure S20. Morphological evaluation of PC-3 cells treated with each compound. PC-3 were treated with vehicle (methanol) and increasing concentrations (0-100 µM) of **Clm-1, Clm-2** and **Clm-3** for 48 h. Results are representative of three independent experiments. Scale bar: 200 µm.

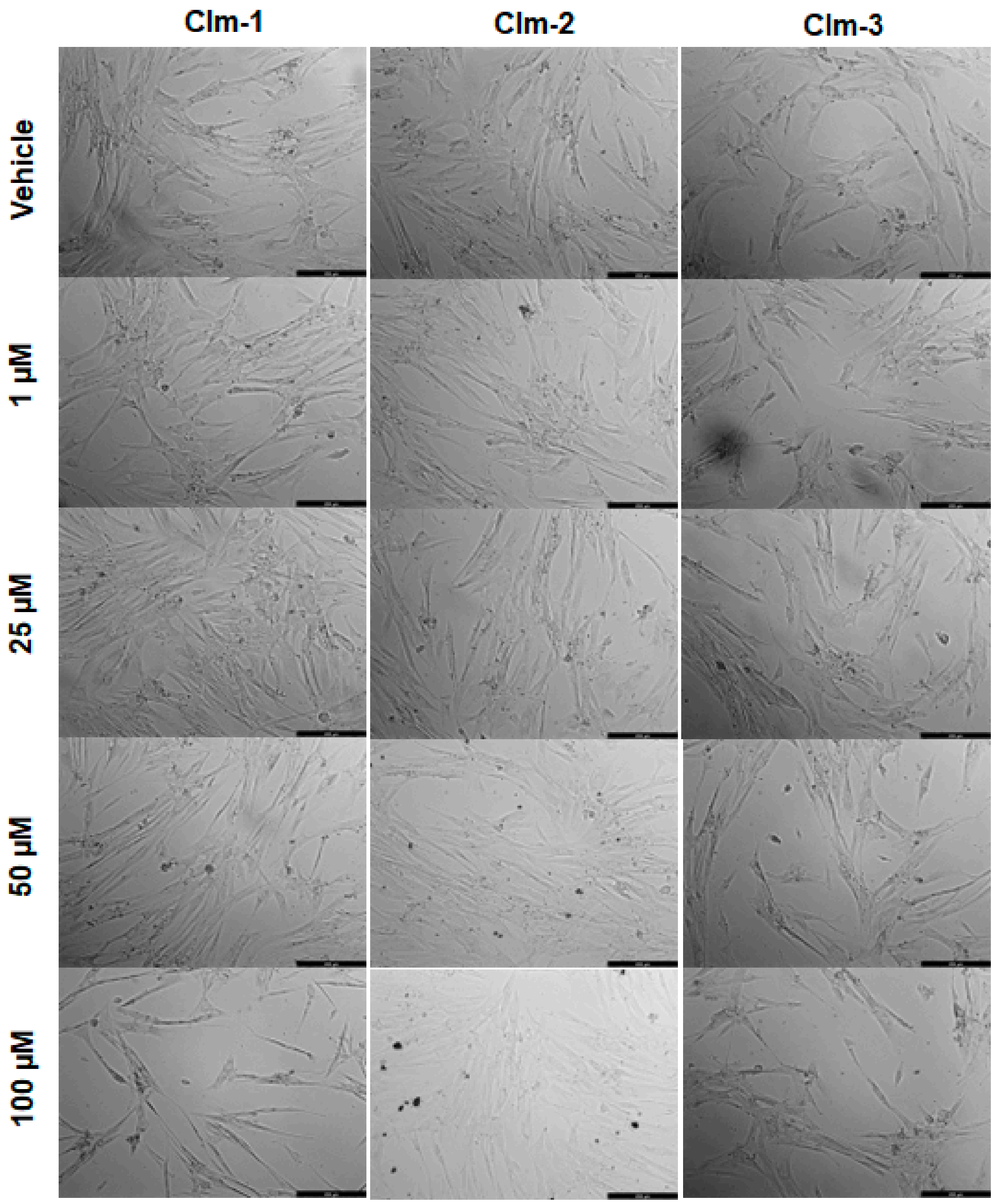

Figure S21. Morphological evaluation of MRC-5 cells treated with each compound. MRC-5 were treated with vehicle (methanol) and increasing concentrations (0-100 µM) of **Clm-1**, **Clm-2** and **Clm-3** for 48 h. Results are representative of three independent experiments. Scale bar: 200 µm.

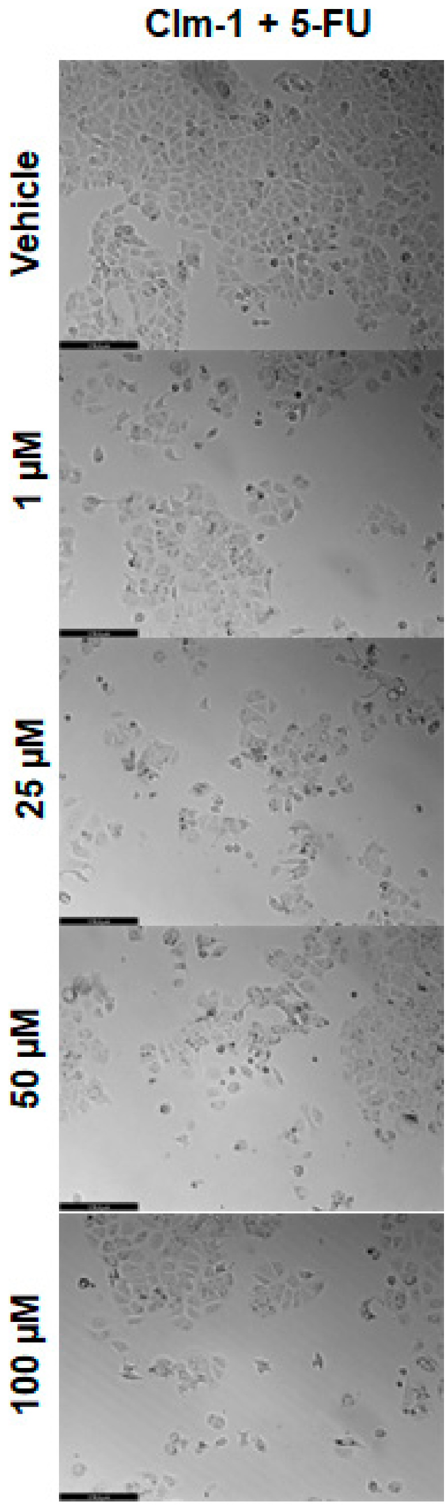

Figure S22. Morphological evaluation of MCF-7 cells treated with a combination of 5-FU (11.79 µM) and different concentrations of C1. MCF-7 were treated with vehicle (methanol and DMSO) and a combination of 11.79 µM of 5-FU and increasing concentrations (0-100 µM) of **Clm-1** for 48 h. Results are representative of three independent experiments. Scale bar: 200 µm.

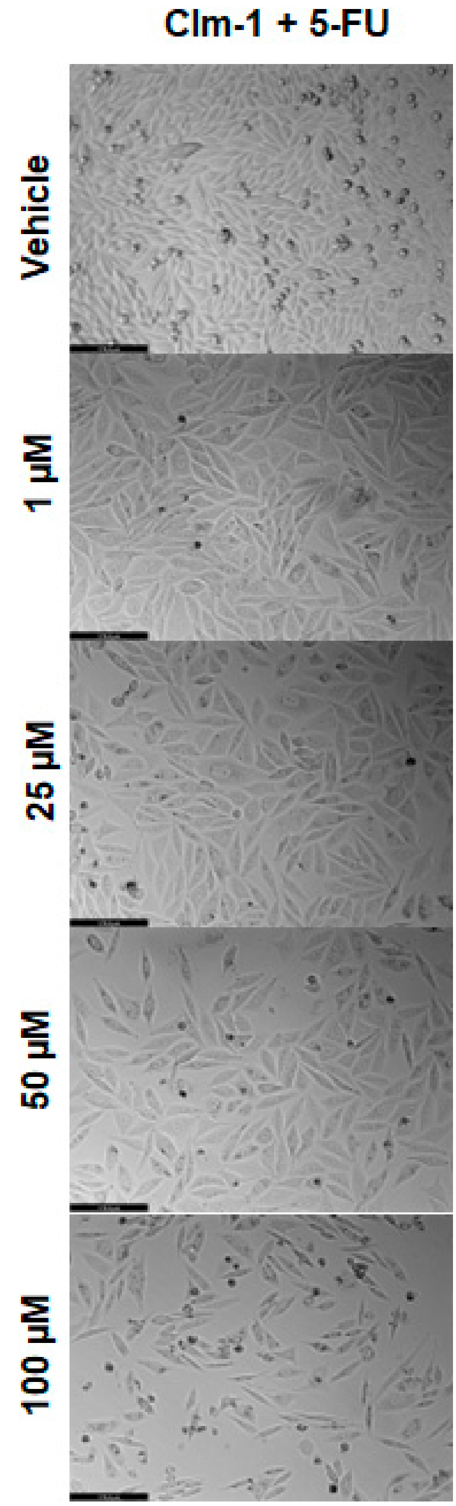

Figure S23. Morphological evaluation of PC-3 cells treated with a combination of 5-FU (11.79 µM) and different concentrations of C1. MCF-7 were treated with vehicle (methanol and DMSO) and a combination of 11.79 µM of 5-FU and increasing concentrations (0-100 µM) of **Clm-1** for 48 h. Results are representative of three independent experiments. Scale bar: 200 µm.
